# Do Ketogenic Diets Produce Greater Weight Loss than Higher-Carbohydrate Diets Under Comparable Prescribed Energy Conditions? A Systematic Review and Meta-Analysis of Randomized Controlled Trials

**DOI:** 10.3390/nu18152525

**Published:** 2026-08-04

**Authors:** Tomasz Żurawski, Anna Bartosiewicz

**Affiliations:** 1 University of Rzeszów, Collegium Medicum, Faculty of Health Sciences and Psychology, 35-959 Rzeszów, Poland; 2 University Center for Research and Development in Health Sciences, 35-310 Rzeszów, Poland

**Keywords:** ketogenic diet, carbohydrate restriction, very-low-carbohydrate diet, low carb, obesity, overweight, weight loss, systematic review, meta-analysis

## Abstract

**Background/Objectives**: Whether ketogenic diets provide greater weight-loss benefits than higher-carbohydrate diets when prescribed energy intake or targeted energy deficit are comparable remains uncertain. This systematic review and meta-analysis evaluated their effects on body weight change in adults with overweight or obesity. **Methods**: Following PRISMA 2020, PubMed, Scopus, and Web of Science were searched for randomized controlled trials comparing ketogenic diets with higher-carbohydrate diets under matched energy prescriptions. Ketogenic diets were defined as providing ≤50 g carbohydrate/day or ≤10% of total energy from carbohydrate. A random-effects meta-analysis used restricted maximum likelihood estimation and the Hartung–Knapp adjustment. Risk of bias and certainty of evidence were assessed using RoB 2 and GRADE, respectively. **Results**: Eight reports representing seven studies were included in the systematic review; six studies comprising 259 participants contributed to the meta-analysis. Ketogenic diets were associated with slightly greater weight loss than higher-carbohydrate diets (MD = −1.49 kg; 95% CI: −2.41 to −0.58; *p* = 0.008; *I*^2^ = 0%). Estimates remained similar after alternative imputations of change-score standard deviations, exclusion of the trial at high risk of bias, and leave-one-out analyses. In three trials meeting the prescribed protein-matching criterion, the effect remained directionally consistent but was imprecise and compatible with no difference. Certainty of evidence was low because of risk of bias and imprecision. **Conclusions**: Under comparable prescribed energy conditions, ketogenic diets may produce a small additional reduction in body weight. However, predominantly short-term evidence, uncertainty regarding achieved energy and protein intakes, the absence of a synthesis of body composition, and low certainty of evidence limit confidence in the magnitude, durability, and clinical significance of this advantage.

## 1. Introduction

Obesity is a chronic, multifactorial disease whose prevalence has increased worldwide over recent decades [1,2]. It is defined as an abnormal or excessive accumulation of adipose tissue that may impair health [3]. Although its development is associated with a sustained positive energy balance [3,4], the course of the disease also depends on interactions among biological, environmental, and behavioral factors [5,6]. Obesity is associated with an increased risk of type 2 diabetes [7,8], cardiovascular disease [9], certain cancers [10], musculoskeletal disorders [11], and premature mortality [12,13]. Reducing excess body weight therefore remains one of the principal goals of treatment, and appropriately planned dietary therapy constitutes a key component of management [14].

Weight loss requires a negative energy balance, in which energy intake remains lower than total energy expenditure [4]. However, energy balance is dynamic, as both energy intake and expenditure may change with weight loss, physical activity level, and dietary composition [14,15]. Various dietary approaches are used in clinical practice, including low-fat, Mediterranean, high-protein, and low-carbohydrate diets [14]. Many of these approaches can lead to weight loss, although differences between individual strategies often diminish as the duration of the intervention increases [14,16,17,18].

In recent years, low-carbohydrate and ketogenic diets have attracted considerable attention. However, the term low-carbohydrate diet encompasses a broad range of interventions that differ substantially in the degree of carbohydrate restriction [19]. In some studies, interventions providing less than 30% of total energy from carbohydrate were classified as low-carbohydrate diets, whereas others applied a threshold of less than 100 g of carbohydrate per day [19]. A ketogenic diet represents a more restrictive form of this approach and is commonly defined as providing ≤50 g of carbohydrate per day or ≤10% of total energy from carbohydrate [20]. However, labeling an intervention as ketogenic does not necessarily indicate that the carbohydrate intake achieved by participants was consistent with the protocol targets or the adopted criteria for a ketogenic diet [20,21]. Differences in definitions and discrepancies between prescribed and achieved intake may therefore lead to misclassification of interventions and hinder comparison across studies.

One concept proposed to explain the possible advantage of low-carbohydrate diets is the carbohydrate–insulin model of obesity [22]. According to this model, a higher carbohydrate intake and the associated insulin response would promote energy storage in adipose tissue [22]. However, the key predictions of this model have not been conclusively confirmed in experimental studies [21,23]. Accordingly, any differences in weight loss should not be attributed solely to the degree of carbohydrate restriction without accounting for energy intake.

Previous analyses have sometimes reported greater short-term weight loss with low-carbohydrate diets than with low-fat or higher-carbohydrate diets [24,25]. However, these differences often diminished with longer follow-up [16,17,18]. Interpretation of the available evidence is complicated by heterogeneity across studies in the degree of carbohydrate restriction, energy intake, protein intake, and intervention duration [26]. The lack of comparable energy prescriptions is particularly important, because greater weight loss in one group may result from a larger prescribed or achieved energy deficit rather than from macronutrient distribution itself. Restricting the analysis to studies in which the compared interventions had the same prescribed energy intake or the same targeted energy deficit may therefore allow a more precise assessment of the comparative effectiveness of ketogenic and higher-carbohydrate diets.

The aim of this systematic review and meta-analysis was to estimate the difference in body weight change between ketogenic and higher-carbohydrate diets in adults with overweight or obesity, based on randomized controlled trials designed to compare the diets under comparable prescribed energy conditions.

## 2. Materials and Methods

### 2.1. Protocol and Reporting Standards

This systematic review and meta-analysis was conducted in accordance with the recommendations of the Cochrane Handbook for Systematic Reviews of Interventions [27]. The manuscript was prepared in accordance with the Preferred Reporting Items for Systematic Reviews and Meta-Analyses 2020 (PRISMA 2020) guidelines [28], and the completed PRISMA 2020 checklist is provided in Supplementary Table S1. The review was prospectively registered in the International Prospective Register of Systematic Reviews (PROSPERO; CRD420251134692) before the formal literature search and study selection were initiated. Deviations from the methods registered in PROSPERO, together with their justifications, are presented in Supplementary Table S2.

### 2.2. Eligibility Criteria

Eligibility criteria were defined a priori according to the Population, Intervention, Comparator, and Outcome (PICO) framework [27] together with prespecified study-design criteria. Eligible studies were randomized controlled trials, including randomized pilot trials with an appropriate comparator group, conducted in adults aged 18–65 years with overweight or obesity. Overweight was defined as a body mass index (BMI) of 25.0–29.9 kg/m^2^, and obesity as a BMI ≥ 30.0 kg/m^2^ [29]. Studies including mixed populations were eligible only if all participants met the criteria for overweight or obesity or if data for the eligible subgroup were reported separately. The presence of metabolic diseases coexisting with excess body weight was not an exclusion criterion.

The eligible intervention was a ketogenic or very-low-carbohydrate diet, defined as providing ≤50 g of carbohydrate per day or ≤10% of total energy intake from carbohydrate [20]. When both prescribed carbohydrate intake and achieved intake during the intervention were reported, eligibility was determined based on the achieved values. Biochemical confirmation of ketosis was not required. The comparator was a higher-carbohydrate diet. Only studies in which the compared interventions had the same prescribed energy intake or the same targeted energy deficit were eligible. Studies in which at least one arm followed an ad libitum diet without a predefined and comparable energy prescription were excluded.

The minimum intervention duration was >4 weeks. Studies were required to report change in body weight as an outcome or provide data from which body-weight change could be derived. Change in body weight could be a primary or secondary outcome. For inclusion in the meta-analysis, body-weight change had to be available in kilograms, or be convertible to kilograms, together with an appropriate measure of dispersion.

Only full-text articles published in English were included. Quasi-randomized and nonrandomized studies, observational studies, studies without an appropriate comparator group, case series and case reports, reviews, meta-analyses, commentaries, letters to the editor, conference abstracts, theses, doctoral dissertations, institutional reports, and other non-full-text or unpublished materials were excluded.

### 2.3. Information Sources and Search Strategy

PubMed, Scopus, and the Web of Science were searched from inception to 30 September 2025. The searches were executed on 9 October 2025 for PubMed and Scopus and on 14 October 2025 for Web of Science. The search was restricted to publications in English. In addition, the reference lists of publications assessed in full text were manually screened to identify potentially eligible studies not retrieved through the electronic searches. No separate search for grey or unpublished literature was conducted.

Two complementary search strategies, adapted to the syntax and indexing structure of each database, were applied in each database. The complete search strings exactly as run, the limits applied, the search dates, and the numbers of records retrieved are presented in Supplementary Table S3.

### 2.4. Study Selection Process

All records identified through the search were imported into Zotero, version 8.0.4 (Corporation for Digital Scholarship, Vienna, VA, USA), which was used for reference management and for the identification and removal of duplicates. Two reviewers (TZ and AB) then independently screened titles and abstracts, followed by the full texts of potentially eligible publications, using the prespecified eligibility criteria. Disagreements were resolved through discussion.

For each publication excluded after full-text assessment, one primary reason for exclusion was recorded. The complete list of excluded publications, together with the corresponding reasons, is provided in Supplementary Table S4.

### 2.5. Data Collection Process and Management of Multiple Reports

Data were extracted using a prespecified form developed in Google Sheets (Google LLC, Mountain View, CA, USA). Data extraction was performed by one reviewer (TZ), and the completeness and consistency of the extracted data with the source publications were verified by a second reviewer (AB). Any discrepancies were resolved through discussion. Data were obtained from the full texts, tables, and Supplementary Materials. When data were missing or unclear, attempts were made to contact the study authors.

Reports relating to the same randomized trial were identified and grouped at the study level. Methodological and outcome information was cross-referenced and supplemented across related publications. When more than one report from the same trial met the eligibility criteria and provided relevant data, these reports were treated in the qualitative synthesis as multiple publications relating to a single study. In the quantitative synthesis, participants were included only once. When data from several eligible time points were available for the same randomized trial, results from the longest available follow-up for which a complete set of data on change in body weight could be obtained were used. This rule also applied to measurements collected after the active intervention phase if participants remained under follow-up within the original randomized comparison.

### 2.6. Data Items and Outcome Definition

Data were extracted regarding study identification and design, country, participant characteristics, eligibility criteria, group sizes at randomization and at the analyzed time point, intervention duration, and the analysis population used for outcome assessment. Information describing the intervention and comparator was also collected, including prescribed and achieved energy and macronutrient intakes, intervention delivery, control of physical activity, methods used to assess adherence, and biochemical confirmation of ketosis, when reported.

The primary outcome was the change in body weight from baseline to the longest eligible time point available within each randomized comparison. For quantitative synthesis, the outcome was expressed in kilograms. This time point could correspond to the end of the active dietary intervention or to a later follow-up assessment, provided that the original randomized group allocation was retained and data were available to estimate the comparative effect. For each study arm, the number of participants with outcome data available at the analyzed time point, the mean change in body weight, and the corresponding measure of dispersion were extracted. Directly reported values for change in body weight were preferred. When data were not presented in the format required for quantitative synthesis, the parameters needed to calculate them were extracted. Data preparation and conversion procedures are described in Section 2.7.

### 2.7. Effect Measure and Data Preparation

The intervention effect was expressed as the mean difference (MD) in body weight change between the ketogenic-diet group and the higher-carbohydrate-diet group. Negative values indicated greater weight loss in the ketogenic-diet group. Because all studies included in the quantitative synthesis reported, or allowed derivation of, body weight change in kilograms, standardized mean differences were not used.

The directly reported mean change in body weight from baseline to the analyzed time point, together with its standard deviation, was preferred. When the mean change was not reported directly, it was calculated as the difference between the final and baseline values. When the standard error of the mean change was reported, the standard deviation was calculated using the following formula:

SD = SE × √n

When the standard deviation of the change was unavailable but standard deviations at baseline and follow-up were reported, it was calculated using the following formula:

SD_change_ = √(SD_baseline_^2^ + SD_final_^2^ − 2r × SD_baseline_ × SD_final_)

where r denotes the correlation coefficient between baseline and follow-up measurements. A value of r = 0.50 was assumed in the primary analysis. The influence of this assumption was assessed in sensitivity analyses using r = 0.25 and r = 0.75.

The quantitative synthesis was based on data from participants for whom body-weight-change outcomes were available at the analyzed time point. Accordingly, the sample sizes used in the meta-analysis corresponded to the completer or available-case analysis populations reported by the authors of the individual studies and did not always reflect the full number of participants originally randomized. When the available information was insufficient to derive the mean change in kilograms and an appropriate measure of dispersion in the format required for meta-analysis, the study was included only in the qualitative synthesis.

The input data used in the primary meta-analysis, together with the source of or method used to derive the standard deviation of the change, are presented in Supplementary Table S5.

### 2.8. Risk of Bias and Certainty of Evidence

Risk of bias for the body-weight-change outcome was assessed independently by two reviewers (TZ and AB) using the Cochrane Risk of Bias 2 (RoB 2) tool for the effect of assignment to intervention [27]. Disagreements were resolved through discussion. The certainty of evidence for the primary outcome was evaluated using the Grading of Recommendations Assessment, Development and Evaluation (GRADE) approach [27], considering risk of bias, inconsistency, indirectness, imprecision, and publication bias.

### 2.9. Statistical Analysis

The meta-analysis was conducted using a random-effects model and the inverse-variance method. Between-study variance was estimated using restricted maximum likelihood (REML), and 95% confidence intervals for the pooled effect were calculated using the Hartung–Knapp adjustment. The pooled effect was expressed as the mean difference (MD) in body weight change with a 95% confidence interval. Statistical heterogeneity was assessed using Cochran’s Q statistic, *I*^2^, and τ^2^. No subgroup analysis or meta-regression was performed because the small number of included studies did not permit a reliable investigation of potential sources of heterogeneity.

The influence of the correlation coefficient assumed when calculating the standard deviation of the change was examined in prespecified sensitivity analyses by repeating the meta-analysis using r = 0.25 and r = 0.75, with r = 0.50 used in the primary analysis.

Three additional post hoc sensitivity analyses were performed. The stability of the pooled effect was assessed using a leave-one-out analysis, in which each study was excluded in turn. An analysis excluding the study rated as having an overall high risk of bias and an analysis restricted to studies in which prescribed protein intake was matched between the compared interventions were also conducted. These analyses were exploratory and were intended to assess the robustness of the primary finding to selected methodological assumptions and characteristics of the included studies.

Statistical analyses were performed in R (R Foundation for Statistical Computing, Vienna, Austria), version 4.5.3, using RStudio (Posit Software, PBC, Boston, MA, USA), version 2026.01.1 (Build 403). Meta-analyses were conducted using the meta package, version 8.2-1. All statistical tests were two-sided, and statistical significance was defined as *p* < 0.05. The input data, complete analytical code, information on the computational environment, and script-generated outputs are provided in the Supplementary Reproducibility File S1.

### 2.10. Assessment of Reporting Bias

Because only a small number of studies were included in the meta-analysis, no formal assessment of funnel-plot asymmetry or tests for small-study effects were performed, as their results would not have allowed a reliable assessment of the risk of publication bias [30].

## 3. Results

### 3.1. Study Selection

The systematic search of PubMed, Scopus, and Web of Science identified a total of 3959 records, including 468 from PubMed, 2088 from Scopus, and 1403 from Web of Science. After removal of 1715 duplicate records, 2244 records remained for title and abstract screening. At this stage, 2026 records were excluded, and full-text retrieval was attempted for 218 reports.

Full texts could not be obtained for 2 reports. Accordingly, 216 reports were assessed for eligibility, of which 208 were excluded after full-text review. The most common reasons for exclusion were failure to meet the intervention criteria (*n* = 77), lack of energy matching between the compared interventions (*n* = 57), an ineligible study design or publication type (*n* = 29), absence of an appropriate comparator group (*n* = 19), and duplication or secondary reporting (*n* = 16). The remaining reports were excluded because of an ineligible population (*n* = 5), insufficient intervention duration (*n* = 3), or absence of eligible outcome data (*n* = 2). A detailed list of reports excluded after full-text assessment, together with the primary reason for exclusion, is provided in Supplementary Table S4.

**Figure 1:**
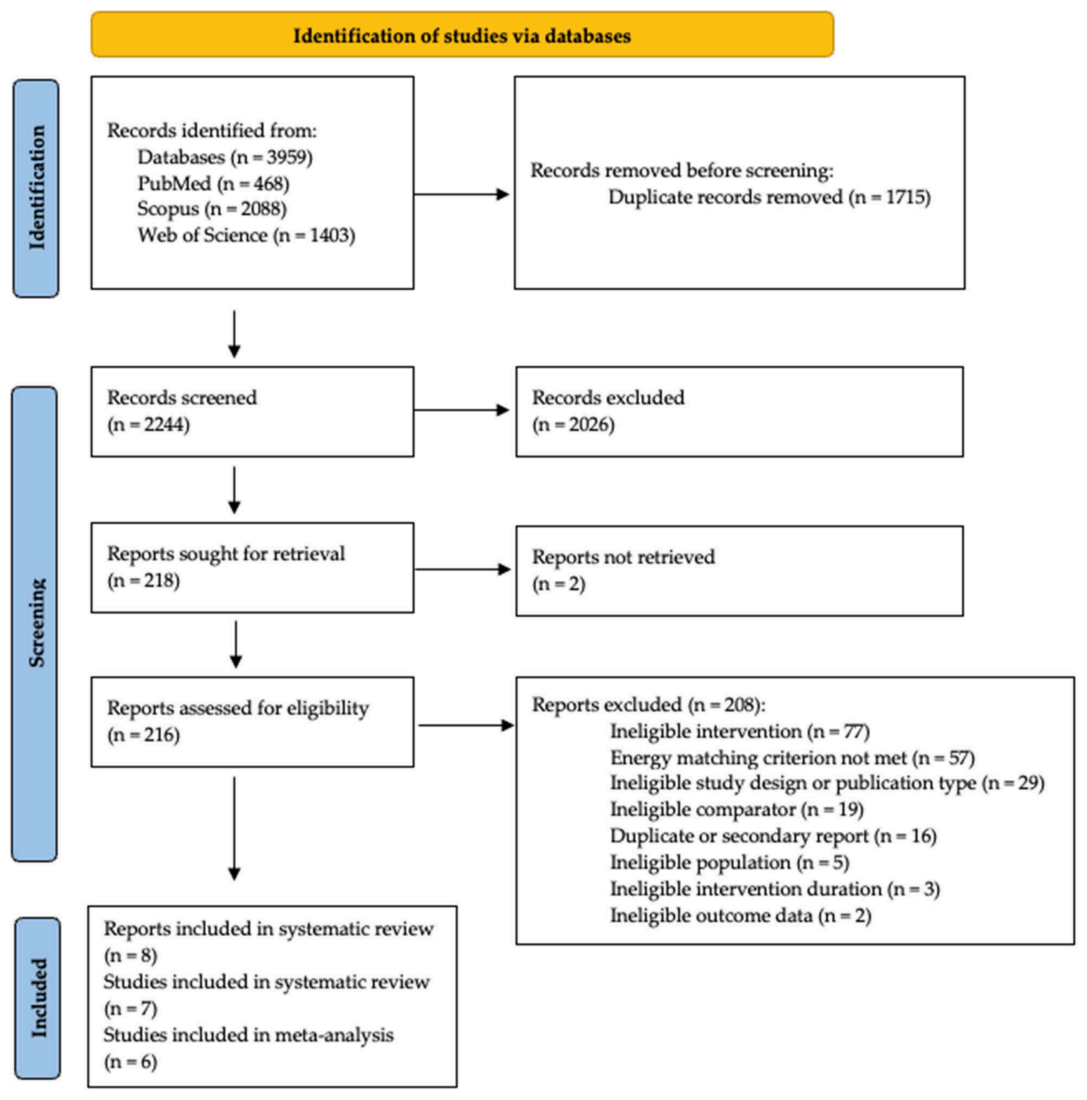
PRISMA 2020 flow diagram of study identification, screening, eligibility assessment, and inclusion.

Ultimately, 8 reports relating to 7 distinct studies were included in the systematic review [31,32,33,34,35,36,37,38]. Six studies provided data suitable for quantitative synthesis and were included in the meta-analysis [31,32,33,34,35,36]. Two reports were only included in the qualitative synthesis [37,38]. One of these related to the same study cohort as a report included in the meta-analysis but presented results from an earlier time point [32,38]. In accordance with the prespecified procedure for multiple reports from the same study, the meta-analysis used data from the report with the longest eligible follow-up period [32]. The other report did not provide data suitable for quantitative synthesis [37]. Despite attempts to contact the authors, the missing data could not be obtained. The study selection process is presented in Figure 1.

### 3.2. Study Characteristics

The included studies were conducted in the United States [36,37], Australia [32,35,38], Switzerland [33], China [34], and Saudi Arabia [31]. Sample sizes at randomization ranged from 28 to 118 participants, and intervention durations ranged from 6 to 52 weeks [31,32,33,34,35,36,37,38]. All studies used a parallel-group design. One study [35] included three dietary intervention arms, but only the comparison between two of these arms was eligible for the present review.

The study populations comprised adults with overweight or obesity, although the specific clinical eligibility criteria varied across trials. Some studies recruited participants without major chronic diseases other than excess body weight [33,36,37]. One study required a BMI > 28 kg/m^2^ and at least one additional cardiovascular risk factor [35], whereas the Australian trial reported in two publications included participants with abdominal obesity and at least one additional risk factor for metabolic syndrome [32,38]. Another study included adults with overweight or obesity and newly diagnosed type 2 diabetes [34], while one trial was conducted exclusively in women aged 18–40 years with overweight or obesity [31].

All interventions met the prespecified definition of a ketogenic diet based on prescribed or achieved carbohydrate intake of ≤50 g/day or ≤10% of total energy intake. In most studies, the target carbohydrate intake ranged from 4% to 10% of total energy [31,32,35,38] or from 20 to 50 g/day [34,36,37]. In one trial, carbohydrates provided 15% of total energy, corresponding to approximately 37 g/day. Therefore, the intervention met the eligibility criterion based on the absolute intake expressed in grams [33]. Comparators were higher-carbohydrate diets, typically providing 45% to 70% of total energy from carbohydrates [31,32,33,35,37,38] or approximately 250–280 g/day when recommendations were expressed in absolute amounts [34].

All studies prescribed the same energy intake or the same targeted energy deficit across the compared groups [31,32,33,34,35,36,37,38]. However, the degree of control over intervention delivery varied. One trial used fully controlled feeding in a research-unit setting [33], whereas another prepared and provided all meals to participants in an outpatient setting [36]. In two Australian trials, selected key food items were provided, while the remaining components of the diet were implemented under free-living conditions [32,35,38]. In the remaining studies, the interventions were based on dietary counseling, nutrition education, meal plans, or self-preparation of meals by participants [31,34,37].

Adherence was assessed using food diaries, weighed dietary records, meal photographs, regular dietitian consultations, monitoring of food collection, and body-weight monitoring [31,32,33,34,35,36,37,38]. Plasma ketones were measured in two Australian trials [32,35,38], while two other studies assessed urinary ketones using reagent strips [31,36]. Two studies did not report biochemical assessment of ketosis [33,37], and in one study the method used to confirm ketosis was not clearly described [34].

The degree of protein matching varied across studies. Three studies met the protocol-defined criterion, which allowed a between-group difference of no more than 0.3 g/kg/day or 5 percentage points of total energy intake [31,33,34]. In one of these studies, protein provided 20% and 25% of total energy in the respective groups, corresponding exactly to the maximum permitted difference of 5 percentage points [31]. In the remaining studies, the ketogenic diet provided a higher proportion of energy from protein than the comparator diet [32,35,36,37,38].

Detailed characteristics of the studies, interventions, and body-weight outcomes are presented in Table 1. Additional information on feeding conditions, methods used to assess adherence and ketosis, and the status of energy and protein matching is provided in Supplementary Table S6.

### 3.3. Effects on Body Weight Loss

**Figure 2:**
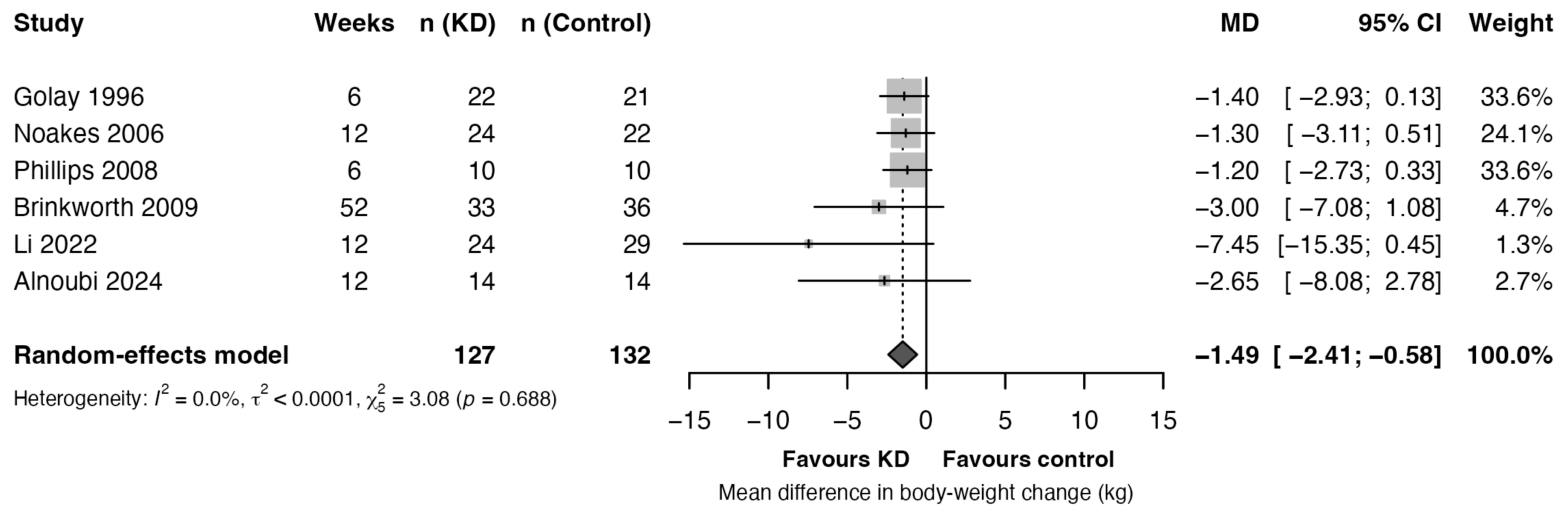
Forest plot of the effect of ketogenic diets versus higher-carbohydrate diets on body weight loss [31,32,33,34,35,36]. Negative mean differences indicate greater body-weight reduction in the ketogenic-diet group. CI, confidence interval; KD, ketogenic diet; MD, mean difference.

Of the seven included studies, six provided data on absolute change in body weight that could be included in the meta-analysis [31,32,33,34,35,36]. The quantitative synthesis included a total of 259 participants, of whom 127 were assigned to ketogenic-diet groups and 132 to higher-carbohydrate-diet groups. Study-specific effect estimates and the pooled meta-analysis result are presented in Figure 2.

In the random-effects model, with between-study variance estimated using REML and confidence intervals calculated using the Hartung–Knapp adjustment, the ketogenic diet was associated with greater weight loss than the higher-carbohydrate diet. The pooled mean difference was −1.49 kg (95% CI: −2.41 to −0.58; *p* = 0.008). No statistically significant heterogeneity was detected across studies (Q = 3.08; *df* = 5; *p* = 0.688), and the estimated between-study variance and heterogeneity were τ^2^ < 0.0001 and *I*^2^ = 0.0%, respectively.

Two included reports were not entered as separate comparisons in the meta-analysis [37,38]. In one report, mean body-weight change was presented only as a percentage of baseline body weight and was −7.1 ± 4.6% in the ketogenic-diet group and −5.3 ± 4.7% in the higher-carbohydrate-diet group [37]. Because data were unavailable to express the effect as an absolute change in kilograms, this report could not be included in the quantitative synthesis. The second report presented 24-week results from an Australian trial, in which mean weight loss was −11.9 ± 6.3 kg in the ketogenic-diet group and −10.1 ± 5.7 kg in the higher-carbohydrate-diet group [38]. Because this report concerned the same cohort as the publication reporting the 52-week follow-up [32], only the outcome from the longest eligible time point was included in the meta-analysis to avoid counting the same participants more than once.

### 3.4. Sensitivity Analyses

The results of analyses based on alternative values of the correlation coefficient used to impute the standard deviation of the change were similar to those of the primary analysis. Assuming r = 0.25, the pooled mean difference was −1.46 kg (95% CI: −2.25 to −0.67; *p* = 0.005; *I*^2^ = 0.0%), whereas assuming r = 0.75, it was −1.59 kg (95% CI: −2.78 to −0.41; *p* = 0.018; *I*^2^ = 6.7%).

After exclusion of the study rated as having a high risk of bias, the estimated effect was −1.42 kg (95% CI: −2.01 to −0.83; *p* = 0.003; *I*^2^ = 0.0%).

Of the six studies included in the meta-analysis, three met the registered criterion for matching prescribed protein intake [31,33,34]. In the exploratory analysis restricted to these studies, the pooled mean difference was −1.79 kg (95% CI: −5.69 to 2.11; *p* = 0.187; *I*^2^ = 13.0%). The confidence interval was wide and included the null value.

In the leave-one-out analysis, the estimated mean differences after sequential exclusion of individual studies ranged from −1.42 to −1.64 kg. In all iterations, the 95% confidence intervals excluded the null value.

The results of all sensitivity analyses are presented in Supplementary Figures S1–S5.

### 3.5. Risk of Bias

None of the seven studies was judged to be at low risk of bias across all domains. Six studies received an overall rating of “some concerns” [31,32,33,35,36,37,38], whereas one study was judged to be at high risk of bias [34]. The high-risk rating in this study was driven primarily by missing outcome data, as the dropout rate was substantially higher in the ketogenic-diet group [34].

**Figure 3:**
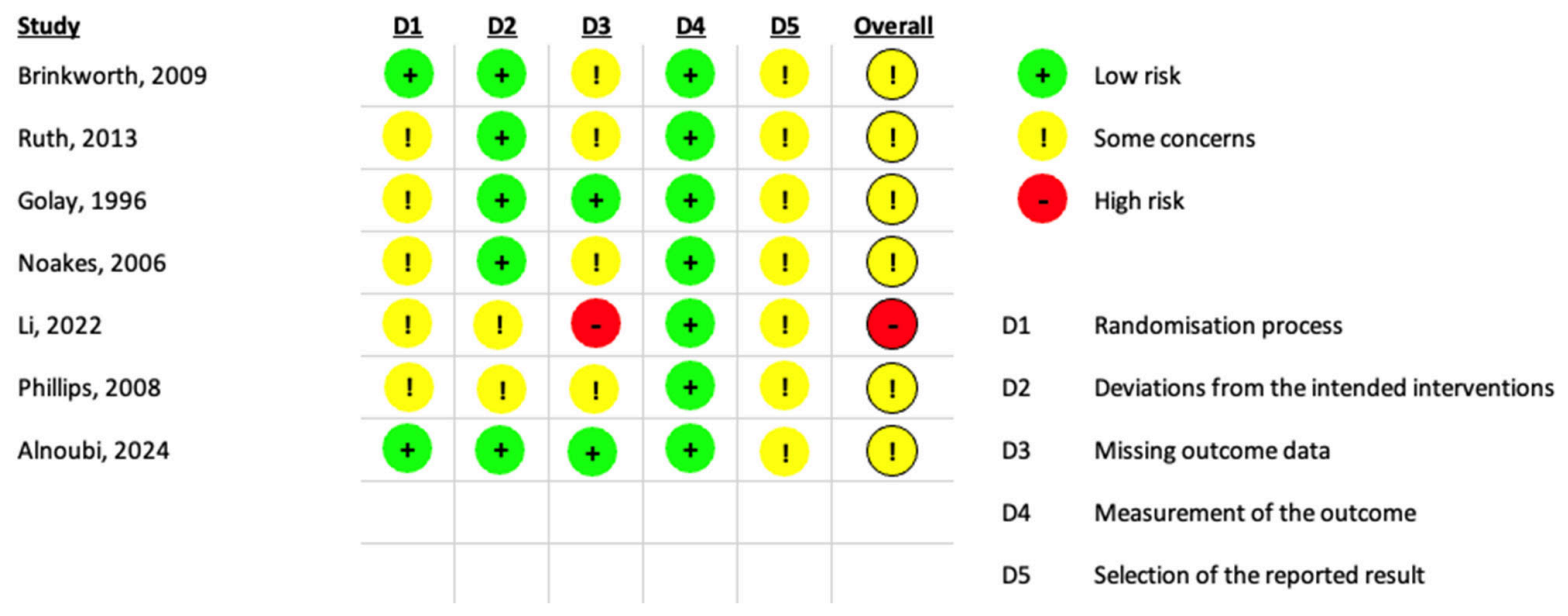
Risk-of-bias assessment for the body-weight outcome using the revised Cochrane risk-of-bias tool for randomized trials (RoB 2) [31,32,33,34,35,36,37]. Assessments refer to the effect of assignment to intervention.

The most common concerns related to insufficient reporting of the randomization process and the possibility of selection among multiple eligible methods for analyzing or reporting change in body weight in the absence of detailed, prospectively specified analysis plans. Concerns regarding missing outcome data were identified in five studies [32,34,35,36,37], whereas measurement of body weight was judged to be at low risk of bias in all studies [31,32,33,34,35,36,37,38]. Domain-level risk-of-bias assessments are presented in Figure 3, and detailed justifications for the judgments are provided in Supplementary Table S7.

### 3.6. Certainty of Evidence

The certainty of evidence for the effect of ketogenic diets, compared with higher-carbohydrate diets, on weight loss was rated as low. The certainty was downgraded by one level for serious concerns regarding risk of bias and by one level for serious imprecision. There was no basis for downgrading the certainty of evidence for inconsistency or indirectness. Because of the small number of studies, publication bias could not be assessed reliably using formal methods. No additional downgrade was applied for publication bias because no specific evidence suggesting selective non-publication or missing results was identified. The detailed assessment is presented in Table 2.

## 4. Discussion

In this systematic review and meta-analysis, ketogenic diets were associated with greater weight loss than higher-carbohydrate diets in adults with overweight or obesity (MD = −1.49 kg; 95% CI: −2.41 to −0.58; *p* = 0.008). The quantitative synthesis was restricted to randomized trials in which the compared groups received the same prescribed energy intake or the same targeted energy deficit. This eligibility criterion reduced the potential influence of differences in planned energy restriction between groups, but it did not establish that achieved energy intake was equivalent throughout the intervention. The pooled estimate remained similar under alternative assumptions used to impute the standard deviation of the change, after exclusion of the study at high risk of bias, and in the leave-one-out analysis. However, the low certainty of evidence, resulting from risk of bias and imprecision, limits confidence in the magnitude of the effect. The result should therefore be interpreted as evidence of a small, predominantly short-term difference in body-weight change under comparable prescribed energy conditions, rather than as evidence of clear clinical superiority.

The direction of the present finding is consistent with some previous syntheses [17,18], although direct comparison of effect sizes requires consideration of differences in intervention definitions, follow-up duration, and energy prescriptions. A meta-analysis restricted to very-low-carbohydrate ketogenic diets with at least 12 months of follow-up reported greater weight loss than low-fat diets, but the mean difference was only −0.91 kg (95% CI: −1.65 to −0.17 kg) [17]. Another meta-analysis reported a larger difference of −2.17 kg (95% CI: −3.36 to −0.99 kg) [18], but used a broader definition of carbohydrate restriction and included predominantly ad libitum low-carbohydrate interventions, whereas comparator groups more often received planned energy restriction. Methodological differences among syntheses are also relevant. A systematic review of reviews found substantial variation in the definitions of low-carbohydrate diets, eligibility criteria, and methodological quality [26]. Meta-analyses rated as critically low quality more often reported larger advantages of low-carbohydrate diets, whereas high-quality syntheses generally found small differences or no difference. Larger participant samples were also associated with smaller estimated effects [26]. These findings caution against interpreting the magnitude of any single pooled estimate independently of the methods used to generate it. Against this background, the present meta-analysis addresses a narrower question by applying a more stringent definition of a ketogenic diet and restricting eligibility according to the prespecified energy criterion. It should therefore be viewed as an estimate obtained under more narrowly defined prescription conditions, not as evidence that macronutrient distribution was the only difference between groups.

Most studies included in the present meta-analysis lasted 6 to 12 weeks [31,33,34,35,36], and only one provided data at 52 weeks [32]. This is particularly important when interpreting substantial carbohydrate restriction, because early body weight reduction may partly reflect depletion of glycogen stores and the water associated with them rather than loss of adipose tissue alone [21,39]. Controlled feeding studies also indicate that the increase in fat oxidation that follows carbohydrate restriction does not necessarily translate into greater body-fat loss [21,39]. The observed difference in body weight between groups does not necessarily reflect a proportionally greater reduction in adipose tissue. Because body composition was not synthesized, the relative contributions of changes in adipose tissue, glycogen, and body water remain uncertain. Although the pooled difference of 1.49 kg was statistically significant, its absolute magnitude was modest. Its clinical relevance should be interpreted cautiously because the clinical benefits of weight reduction are generally considered in relation to baseline body weight and associated metabolic outcomes [40], whereas the present result represents an absolute mean difference between groups. The predominance of short interventions, the absence of a synthesis of body composition, and the low certainty of evidence further limit inference about whether this difference represents a durable clinical benefit.

Attribution of the pooled effect is further complicated by differences in protein intake and in the implementation of the interventions. Only three of the six studies met the registered criterion for matching prescribed protein intake [31,33,34]. In the remaining studies, the ketogenic diet provided a higher proportion of energy from protein than the comparator diet [32,35,36]. Higher protein intake may increase satiety and diet-induced thermogenesis and may help preserve fat-free mass during energy restriction [14,41]. These effects can influence spontaneous energy intake and the composition of weight loss even when the prescribed energy targets are comparable. In our exploratory analysis restricted to the three studies meeting the protein-matching criterion, the direction of effect remained consistent with the primary analysis, but the estimate was imprecise and compatible with no difference (MD = −1.79 kg; 95% CI: −5.69 to 2.11). Therefore, this analysis neither confirms nor excludes an independent effect of carbohydrate restriction when protein intake is matched. Intervention delivery also varied substantially. One trial used fully controlled feeding in a research unit [33], and another prepared and provided all meals in an outpatient setting [36], whereas the remaining trials were conducted at least partly under free-living conditions [31,32,34,35]. In those settings, achieved energy and macronutrient intakes were assessed mainly using self-reported methods and may not have remained consistent with the prescriptions throughout follow-up. Consequently, the prespecified energy criterion strengthens control over planned energy exposure, but the pooled effect cannot be attributed with confidence to carbohydrate restriction alone.

From a clinical perspective, a ketogenic diet may represent one possible strategy for weight loss, but its use should be considered individually. Relevant factors not only include the expected change in body weight, but also patient preferences, the feasibility of long-term adherence, tolerability, and coexisting metabolic disorders [14]. Potential benefits may include improved satiety control during weight loss [42] and short-term improvements in glycemic control in individuals with impaired glucose metabolism [43,44]. These benefits should not be regarded as universal or considered in isolation from the individual’s overall metabolic profile. In some studies, ketogenic diets were associated with increases in LDL cholesterol concentrations [17,18], which is clinically relevant given the established causal role of LDL-C in atherosclerotic cardiovascular disease [45]. The use of ketogenic diets should therefore include assessment and monitoring of the lipid profile, particularly in individuals at elevated cardiovascular risk. Diet quality is also important, because substantial carbohydrate restriction may reduce the intake of foods that provide dietary fiber and other bioactive compounds, thereby affecting gastrointestinal function and the composition of the gut microbiota [46]. A ketogenic diet should therefore be regarded neither as inherently optimal nor as inherently unfavorable. It is more appropriately viewed as one therapeutic option whose usefulness depends on the clinical context, the quality of implementation, and the individual response.

Future research requires complementary randomized controlled trials. First, longer, tightly controlled feeding trials are needed, in which energy and protein provision are matched between groups and actual intake is objectively verified, to better isolate the biological effect of carbohydrate restriction. Such studies should include repeated assessments of body composition and objective measures of dietary adherence, including ketone measurements, to distinguish changes in body fat from transient changes in glycogen and body water. Second, adequately powered, larger-scale, randomized controlled trials with longer follow-up periods are needed, conducted under comparable energy conditions in a free-living setting. These studies should assess the feasibility of sustained adherence, weight loss maintenance, and long-term clinical outcomes. Future studies should also systematically document achieved energy and macronutrient intakes throughout the entire follow-up period. In addition to body weight and body composition, relevant outcomes include lipid profile, glycemic control, adverse events, diet quality, and sustained adherence. Together, these two types of evidence would help distinguish the biological effect of dietary composition under strictly controlled conditions from the long-term effectiveness and clinical consequences of using the diet in practice.

## 5. Strengths and Limitations

The strengths of the present review include prospective protocol registration, clearly defined eligibility criteria, and restriction of the quantitative synthesis to randomized trials in which the compared groups received the same prescribed energy intake or the same targeted energy deficit. A ketogenic diet was defined as providing ≤50 g of carbohydrate per day or ≤10% of total energy from carbohydrate. When data on achieved carbohydrate intake were available, they were used to verify whether this criterion had been met, thereby reducing the risk of classifying as ketogenic interventions that did not achieve the specified degree of carbohydrate restriction. The review also used a random-effects model with REML estimation and the Hartung–Knapp adjustment, prespecified and post hoc sensitivity analyses, RoB 2, and GRADE. Consistent management of overlapping reports from the same cohorts reduced the risk of including the same participants more than once.

Several limitations concern the size, duration, and internal validity of the evidence base. The synthesis included six studies and 259 participants, and most interventions lasted 6 to 12 weeks, with only one study providing data at 52 weeks. This limits the precision of the estimate and the ability to assess durability. None of the studies were judged to be at low risk of bias across all domains, and one was classified as being at high risk of bias. Several analyses were based on participants who completed the intervention, increasing susceptibility to bias arising from missing outcome data. Although statistical heterogeneity was not detected, the trials differed in duration, population characteristics, intervention delivery, degree of dietary control, and adherence. An *I*^2^ value of 0% should therefore not be interpreted as evidence of complete clinical homogeneity, particularly given the small number of studies and the limited power of heterogeneity tests. The included trials also enrolled clinically diverse and relatively selected populations, including otherwise healthy adults with obesity, women-only samples, and adults with newly diagnosed type 2 diabetes. Given the small evidence base, it remains uncertain whether the pooled effect is equally applicable across populations with different demographic or clinical characteristics.

Additional limitations concern intervention measurement and the scope of the synthesis. The eligibility criterion addressed prescribed energy intake or targeted energy deficit and did not confirm equivalent achieved energy intake between groups throughout follow-up. Achieved energy and macronutrient intakes were most often assessed using self-reported methods and were not always reported in sufficient detail to confirm maintenance of the intervention targets. Only three studies met the registered protein-matching criterion, and objective indicators of ketosis and adherence were used inconsistently. In some studies, the standard deviation of the change in body weight had to be imputed. Although estimates were similar under alternative correlation assumptions, this remains an additional source of uncertainty. The synthesis focused on body weight change and did not assess body composition, maintenance of weight loss after the intervention, or a comprehensive range of metabolic safety outcomes. The small number of studies also precluded a reliable formal assessment of funnel-plot asymmetry or small-study effects, so publication bias cannot be excluded. Collectively, these limitations resulted in low certainty of evidence. The pooled estimate should therefore be regarded as low-certainty evidence of a small, predominantly short-term difference in body weight change under comparable prescribed energy conditions. It does not establish greater loss of adipose tissue or sustained clinical superiority.

## 6. Conclusions

In this systematic review and meta-analysis, ketogenic diets were associated with slightly greater weight loss than higher-carbohydrate diets in adults with overweight or obesity when the compared groups received the same prescribed energy intake or the same targeted energy deficit. The pooled mean difference was −1.49 kg (95% CI: −2.41 to −0.58; *p* = 0.008). However, the certainty of evidence was rated as low due to the risk of bias and imprecision. The difference between groups was statistically significant, but its magnitude was small, and the preponderance of short-term studies warrants caution in drawing conclusions about the durability or clinical significance of this advantage. Because body composition was not synthesized, the observed difference does not necessarily reflect a proportionally greater reduction in body fat. The findings do not support considering a ketogenic diet to be a universally more effective strategy but indicate that it may represent one possible option for weight loss in appropriately selected individuals. Future studies should include larger samples and longer follow-up, consistently match energy and protein intake, report achieved intake and objective indicators of adherence, and assess body composition, durability of weight loss, and a broader range of metabolic safety outcomes.

## Figures and Tables

**Table 1:** Characteristics and body-weight outcomes of studies included in the systematic review and meta-analysis.

Study and Report	Country and Design	Population	Randomized/Analyzed (*n*)	Duration (Weeks)	Ketogenic or Very-Low-Carbohydrate Diet	Higher-Carbohydrate Comparator	Energy Prescription and Matching	Body-Weight Outcome, as Reported	Included in Meta-Analysis
Golay, 1996 [33]	Switzerland; parallel-group controlled-feeding RCT	Adults with obesity and BMI > 30 kg/m^2^	LC: 22/22; HC: 21/21	6	15% energy from CHO, approximately 37 ± 5 g/day; 32% protein; 53% fat	45% energy from CHO, approximately 115 ± 14 g/day; 29% protein; 26% fat	Fixed intake of 1000 kcal/day in both groups; all food provided and consumed under supervision in a research unit	LC: −8.9 ± 0.6 kg; HC: −7.5 ± 0.5 kg, mean ± SEM	Yes, 6 weeks
Noakes, 2006 [35]	Australia; three-arm, parallel-group RCT	Adults with BMI > 28 kg/m^2^ and at least one additional cardiovascular risk factor	VLCARB: 28/24; VLF: 28/22 ^1^	12	4% energy from CHO, 35% protein, 61% fat; target <20 g CHO/day	70% energy from CHO, 20% protein, 10% fat	Diets designed to be isoenergetic; approximately 30% energy restriction for 8 weeks followed by 4 weeks of energy balance	VLCARB: −8.0 ± 0.6 kg; VLF: −6.7 ± 0.7 kg, mean ± SEM	Yes, 12 weeks
Phillips, 2008 [36]	USA; parallel-group, controlled-feeding RCT	Otherwise healthy adults with obesity, BMI 29–39 kg/m^2^	Total randomized: 28; analyzed: LC 10, LF 10 ^2^	6	Atkins-style diet providing 20 g CHO/day	AHA-style low-fat diet providing 30% of energy from fat	Identical individualized deficit of 750 kcal/day for 4 weeks, followed by 2 weeks of weight stabilization; all meals provided	LC: −5.2 ± 0.6 kg; LF: −4.0 ± 0.5 kg, mean ± SE	Yes, 6 weeks
Tay, 2008 [38]; Brinkworth, 2009 [32] ^3^	Australia; parallel-group RCT with reports at 24 and 52 weeks	Adults with abdominal obesity and at least one additional metabolic syndrome risk factor	Randomized: LC 61, HC 57; analyzed at 24 weeks: LC 45, HC 43 [38]; completers at 52 weeks: LC 33, HC 36 [32]	24 and 52	4% energy from CHO, 35% protein, 61% fat; <20 g CHO/day initially, with allowance up to <40 g/day thereafter	46% energy from CHO, 24% protein, 30% fat	Planned isoenergetic intake of approximately 6 MJ/day for women and 7 MJ/day for men, corresponding to an approximately 30% energy deficit	24 weeks [38]: LC: −11.9 ± 6.3 kg; HC: −10.1 ± 5.7 kg, mean ± SD. 52 weeks [32]: LC: −14.5 ± 1.7 kg; HC: −11.5 ± 1.2 kg, mean ± SEM	Yes, 52-week report [32]
Ruth, 2013 [37]	USA; parallel-group RCT	Free-living adults with obesity, BMI 29.0–44.6 kg/m^2^	HFLC: 29/18; LFHC: 26/15	12	≤40 g CHO/day; approximately 60% energy from fat and 35% from protein	Approximately 60% energy from CHO, 25% fat, and 15% protein	Identical individualized energy deficit of 500 kcal/day in both groups	HFLC: −7.1 ± 4.6%; LFHC: −5.3 ± 4.7% of baseline body weight, mean ± SD	No, outcome unavailable as change in kg
Li, 2022 [34]	China; parallel-group RCT	Adults with overweight or obesity and newly diagnosed type 2 diabetes	KD: 30/24; control: 30/29	12	30–50 g CHO/day, 60 g protein/day, 130 g fat/day	Routine diabetes diet providing 250–280 g CHO/day, 60 g protein/day, and 20 g fat/day	1500 ± 50 kcal/day prescribed in both groups	KD: −8.06 kg; control: −0.61 kg; variability of change not reported	Yes, 12 weeks
Alnoubi, 2024 [31]	Saudi Arabia; parallel-group, single-blind RCT	Healthy women aged 18–40 years with overweight or obesity	HFKD: 14/14; LFD: 14/14	12	10% energy from CHO, 20% protein, 70% fat	55% energy from CHO, 25% protein, 20% fat	Equal individualized total energy prescribed in both groups; numerical energy targets not reported	HFKD: 76.9 ± 8.5 to 66.54 ± 7.7 kg, mean change −10.36 kg; LFD: 73.11 ± 6.7 to 65.4 ± 6.1 kg, mean change −7.71 kg; baseline and follow-up values are mean ± SD	Yes, 12 weeks

^1^ Only the VLCARB and VLF groups were eligible for the present review. The third high-unsaturated-fat group was not included. ^2^ The publication reported 28 participants that were randomized and 20 participants included in the final analysis but did not provide an unambiguous group-specific distribution of all randomized participants. ^3^ Tay et al. [38] and Brinkworth et al. [32] reported outcomes from the same randomized cohort at 24 and 52 weeks, respectively. The 52-week report [32] was used in the meta-analysis in accordance with the prespecified rule to select the longest eligible follow-up. Values in the body-weight outcome column are presented in the units and format reported in the original publications. Calculated or imputed SDs used for the quantitative synthesis are reported separately in Supplementary Table S5. Negative values indicate body weight loss. For Alnoubi and Alqurashi [31], protein prescriptions differed by 5 percentage points of total energy and therefore fell within the tolerance specified in the registered protocol, although protein intake was not identical between groups. Abbreviations: AHA, American Heart Association; CHO, carbohydrate; HC, higher-carbohydrate diet; HFLC, high-fat low-carbohydrate diet; HFKD, high-fat ketogenic diet; KD, ketogenic diet; LC, low-carbohydrate diet; LF, low-fat diet; LFHC, low-fat high-carbohydrate diet; LFD, low-fat diet; RCT, randomized controlled trial; SD, standard deviation; SE, standard error; SEM, standard error of the mean; VLF, very-low-fat diet; VLCARB, very-low-carbohydrate diet.

**Table 2:** Summary of findings for ketogenic diets compared with higher-carbohydrate diets for change in body weight.

Outcome	Participants (Studies)	Effect Estimate	Certainty of the Evidence	Interpretation
Body weight loss, follow-up 6–52 weeks	259 participants (6 RCTs)	MD 1.49 kg greater body weight loss with the ketogenic diet (95% CI: 0.58 to 2.41 kg greater)	⨁⨁◯◯ Low ^a,b^	Ketogenic diets may result in greater body-weight loss than higher-carbohydrate diets under comparable prescribed energy conditions, but the magnitude of the difference remains uncertain.

^a^ Downgraded one level for serious risk of bias. Five studies were rated as having some concerns and one as having high risk of bias. Concerns related primarily to missing outcome data, incomplete reporting of randomization and allocation concealment, and the absence of sufficiently detailed prespecified analysis plans. Excluding the study at high risk of bias yielded a similar pooled estimate. ^b^ Downgraded one level for serious imprecision. The evidence was based on six relatively small trials, and the 95% confidence interval included effects ranging from a small difference to a more appreciable benefit. ⨁⨁◯◯, low certainty of the evidence according to GRADE; CI, confidence interval; MD, mean difference; RCT, randomized controlled trial.

## Data Availability

The study-level data used in the meta-analysis, the complete R code for the primary and sensitivity analyses, information on the computational environment, and script-generated outputs are provided in Supplementary Reproducibility File S1. All data were derived from the published studies cited in this article.

## Supplementary Materials

The following supporting information can be downloaded at https://www.mdpi.com/article/10.3390/nu18152525/s1: Table S1: PRISMA 2020 checklist; Table S2: Deviations from the PROSPERO-registered methods and their rationale; Table S3: Full electronic search strategies; Table S4: Reports excluded after full-text assessment and reasons for exclusion; Table S5: Study-level data used in the primary meta-analysis; Table S6: Detailed characteristics of dietary interventions and assessment of intervention fidelity; Table S7: Risk-of-bias assessments for the body-weight outcome using the RoB 2 tool; Figure S1: Forest plot of the sensitivity analysis assuming a within-participant correlation coefficient of r = 0.25; Figure S2: Forest plot of the sensitivity analysis assuming a within-participant correlation coefficient of r = 0.75; Figure S3: Forest plot of the sensitivity analysis excluding the study rated as being at overall high risk of bias; Figure S4: Forest plot of the exploratory sensitivity analysis restricted to studies meeting the prespecified protein-matching criterion; Figure S5: Leave-one-out sensitivity analysis of the pooled effect on body-weight loss; Supplementary Reproducibility File S1: Study-level analytical data.

## Abbreviations

The following abbreviations are used in this manuscript:

BMI	body mass index
CI	confidence interval
GRADE	Grading of Recommendations Assessment, Development and Evaluation
LDL-C	low-density lipoprotein cholesterol
MD	mean difference
PICO	Population, Intervention, Comparator, and Outcome
PRISMA	Preferred Reporting Items for Systematic Reviews and Meta-Analyses
PROSPERO	International Prospective Register of Systematic Reviews
RCT	randomized controlled trial
REML	restricted maximum likelihood
RoB 2	Cochrane Risk of Bias 2 tool
SD	standard deviation
SE	standard error
SEM	standard error of the mean
