# Supplementary material for: Do Ketogenic Diets Produce Greater Weight Loss than Higher-Carbohydrate Diets Under Comparable Prescribed Energy Conditions? A Systematic Review and Meta-Analysis of Randomized Controlled Trials
Source: Nutrients. 2026 Aug 4;18(15):2525. doi: 10.3390/nu18152525 (PMC13468603; doi:10.3390/nu18152525)
Supplement: Supplementary file 1 [file nutrients-18-02525-s001.zip › nutrients-4454719-supplementary.pdf]

Do Ketogenic Diets Produce Greater Weight Loss than Higher-Carbohydrate Diets Under Comparable Prescribed Energy Conditions? A Systematic Review and Meta-Analysis of Randomized Controlled Trials

Supplementary Materials

CONTENTS

SUPPLEMENTARY TABLES 1

SUPPLEMENTARY TABLE S1. PRISMA 2020 CHECKLIST 1

SUPPLEMENTARY TABLE S2. DEVIATIONS FROM THE PROSPERO-REGISTERED METHODS AND THEIR RATIONALE 5

SUPPLEMENTARY TABLE S3. FULL ELECTRONIC SEARCH STRATEGIES 6

SUPPLEMENTARY TABLE S4. REPORTS EXCLUDED AFTER FULL-TEXT ASSESSMENT AND REASONS FOR EXCLUSION 9

SUPPLEMENTARY TABLE S5. STUDY-LEVEL DATA USED IN THE PRIMARY META-ANALYSIS 32

SUPPLEMENTARY TABLE S6. DETAILED CHARACTERISTICS OF DIETARY INTERVENTIONS AND ASSESSMENT OF INTERVENTION FIDELITY. 33

SUPPLEMENTARY TABLE S7. RISK-OF-BIAS ASSESSMENTS FOR THE BODY-WEIGHT OUTCOME USING THE ROB 2 TOOL. 37

SUPPLEMENTARY FIGURES 40

SUPPLEMENTARY FIGURE S1. FOREST PLOT OF THE SENSITIVITY ANALYSIS ASSUMING A WITHIN-PARTICIPANT CORRELATION COEFFICIENT OF R = 0.25. 40

SUPPLEMENTARY FIGURE S2. FOREST PLOT OF THE SENSITIVITY ANALYSIS ASSUMING A WITHIN-PARTICIPANT CORRELATION COEFFICIENT OF R = 0.75. 40

SUPPLEMENTARY FIGURE S3. FOREST PLOT OF THE SENSITIVITY ANALYSIS EXCLUDING THE STUDY RATED AS BEING AT OVERALL HIGH RISK OF BIAS. 41

SUPPLEMENTARY FIGURE S4. FOREST PLOT OF THE EXPLORATORY SENSITIVITY ANALYSIS RESTRICTED TO STUDIES MEETING THE PRESPECIFIED PROTEIN-MATCHING CRITERION. 41

SUPPLEMENTARY FIGURE S5. LEAVE-ONE-OUT SENSITIVITY ANALYSIS OF THE POOLED EFFECT ON BODY WEIGHT LOSS. 42

Supplementary Tables

Supplementary Table S1. PRISMA 2020 Checklist

Section and Topic	Item #	Checklist item	Location where item is reported
**TITLE**			
Title	1	Identify the report as a systematic review.	Title
**ABSTRACT**			
Abstract	2	See the PRISMA 2020 for Abstracts checklist.	Abstract
**INTRODUCTION**			
Rationale	3	Describe the rationale for the review in the context of existing knowledge.	Introduction
Objectives	4	Provide an explicit statement of the objective(s) or question(s) the review addresses.	Introduction, final paragraph
**METHODS**			
Eligibility criteria	5	Specify the inclusion and exclusion criteria for the review and how studies were grouped for the syntheses.	Section 2.2
Information sources	6	Specify all databases, registers, websites, organisations, reference lists and other sources searched or consulted to identify studies. Specify the date when each source was last searched or consulted.	Section 2.3
Search strategy	7	Present the full search strategies for all databases, registers and websites, including any filters and limits used.	Section 2.3; Supplementary Table S3
Selection process	8	Specify the methods used to decide whether a study met the inclusion criteria of the review, including how many reviewers screened each record and each report retrieved, whether they worked independently, and if applicable, details of automation tools used in the process.	Section 2.4
Data collection process	9	Specify the methods used to collect data from reports, including how many reviewers collected data from each report, whether they worked independently, any processes for obtaining or confirming data from study investigators, and if applicable, details of automation tools used in the process.	Section 2.5
Data items	10a	List and define all outcomes for which data were sought. Specify whether all results that were compatible with each	Section 2.6

		outcome domain in each study were sought (e.g. for all measures, time points, analyses), and if not, the methods used to decide which results to collect.	
	10b	List and define all other variables for which data were sought (e.g. participant and intervention characteristics, funding sources). Describe any assumptions made about any missing or unclear information.	Section 2.6
Study risk of bias assessment	11	Specify the methods used to assess risk of bias in the included studies, including details of the tool(s) used, how many reviewers assessed each study and whether they worked independently, and if applicable, details of automation tools used in the process.	Section 2.8
Effect measures	12	Specify for each outcome the effect measure(s) (e.g. risk ratio, mean difference) used in the synthesis or presentation of results.	Section 2.7
Synthesis methods	13a	Describe the processes used to decide which studies were eligible for each synthesis (e.g. tabulating the study intervention characteristics and comparing against the planned groups for each synthesis (item #5)).	Section 2.2, 2.5, 2.6
	13b	Describe any methods required to prepare the data for presentation or synthesis, such as handling of missing summary statistics, or data conversions.	Section 2.7
	13c	Describe any methods used to tabulate or visually display results of individual studies and syntheses.	Sections 2.6, 2.7, and 2.9; Table 1; Figures 2 and 3
	13d	Describe any methods used to synthesize results and provide a rationale for the choice(s). If meta-analysis was performed, describe the model(s), method(s) to identify the presence and extent of statistical heterogeneity, and software package(s) used.	Section 2.9
	13e	Describe any methods used to explore possible causes of heterogeneity among study results (e.g. subgroup analysis, meta-regression).	Section 2.9, Supplementary Table S2
	13f	Describe any sensitivity analyses conducted to assess robustness of the synthesized results.	Section 2.9 and 2.10
Reporting bias assessment	14	Describe any methods used to assess risk of bias due to missing results in a synthesis (arising from reporting biases).	Section 2.10
Certainty assessment	15	Describe any methods used to assess certainty (or confidence) in the body of evidence for an outcome.	Section 2.8
**RESULTS**			
Study selection	16a	Describe the results of the search and selection process, from the number of records identified in the search to the number of studies included in the review, ideally using a flow diagram.	Section 3.1, Figure 1
	16b	Cite studies that might appear to meet the inclusion criteria, but which were excluded, and explain why they were excluded.	Section 3.1, Supplementary Table S4

Study characteristics	17	Cite each included study and present its characteristics.	Section 3.2; Table 1; Supplementary Table S6
Risk of bias in studies	18	Present assessments of risk of bias for each included study.	Section 3.5, Figure 3, Supplementary Table S7
Results of individual studies	19	For all outcomes, present, for each study: (a) summary statistics for each group (where appropriate) and (b) an effect estimate and its precision (e.g. confidence/credible interval), ideally using structured tables or plots.	Table 1, Section 3.3 and Figure 2
Results of syntheses	20a	For each synthesis, briefly summarise the characteristics and risk of bias among contributing studies.	Sections 3.2 and 3.5; Table 1; Figure 3
	20b	Present results of all statistical syntheses conducted. If meta-analysis was done, present for each the summary estimate and its precision (e.g. confidence/credible interval) and measures of statistical heterogeneity. If comparing groups, describe the direction of the effect.	Sections 3.3 and 3.4; Figure 2; Supplementary Figures S1-S5
	20c	Present results of all investigations of possible causes of heterogeneity among study results.	Not applicable; no formal investigation of potential causes of heterogeneity, such as subgroup analysis or meta-regression, was performed because of the small number of included studies. The exploratory analysis restricted to protein-matched studies was treated as a sensitivity analysis and is reported under Item 20d. See Section 2.9, Section 3.4, and Supplementary

			Table S2.
	20d	Present results of all sensitivity analyses conducted to assess the robustness of the synthesized results.	Section 3.4; Supplementary Figures S1-S5
Reporting biases	21	Present assessments of risk of bias due to missing results (arising from reporting biases) for each synthesis assessed.	Sections 2.10, 3.6, and 5
Certainty of evidence	22	Present assessments of certainty (or confidence) in the body of evidence for each outcome assessed.	Section 3.6; Table 2
**DISCUSSION**			
Discussion	23a	Provide a general interpretation of the results in the context of other evidence.	Section 4
	23b	Discuss any limitations of the evidence included in the review.	Section 4 and 5
	23c	Discuss any limitations of the review processes used.	Section 5
	23d	Discuss implications of the results for practice, policy, and future research.	Section 4 and 6
**OTHER INFORMATION**			
Registration and protocol	24a	Provide registration information for the review, including register name and registration number, or state that the review was not registered.	Section 2.1
	24b	Indicate where the review protocol can be accessed, or state that a protocol was not prepared.	Section 2.1; PROSPERO record CRD420251134692
	24c	Describe and explain any amendments to information provided at registration or in the protocol.	Section 2.1; Supplementary Table S2
Support	25	Describe sources of financial or non-financial support for the review, and the role of the funders or sponsors in the review.	Funding statement
Competing interests	26	Declare any competing interests of review authors.	Conflicts of Interest statement
Availability of data, code and other materials	27	Report which of the following are publicly available and where they can be found: template data collection forms; data extracted from included studies; data used for all analyses; analytic code; any other materials used in the review.	Section 2.9; Data Availability Statement; Supplementary Reproducibility File S1

Supplementary Table S2. Deviations from the PROSPERO-Registered Methods and Their Rationale

Methodological Domain	PROSPERO-Registered Method	Method Applied in the Review	Rationale	Potential Impact on the Review
Protein matching	Eligible comparisons were required to have a between-group difference in protein intake of ≤0.3 g/kg/day or ≤5 percentage points of total energy intake.	Protein matching was not required for inclusion in the primary analysis. A sensitivity analysis was performed using only trials in which protein intake was matched between groups according to the prespecified criterion.	Retaining protein matching as a mandatory eligibility criterion would have excluded several otherwise eligible energy-matched trials and substantially reduced the available evidence base. The sensitivity analysis was therefore used to assess the robustness of the pooled estimate when the analysis was restricted to protein-matched comparisons.	The primary analysis included trials with possible between-group differences in protein intake. In the protein-matched sensitivity analysis, the direction of the effect remained consistent with the primary analysis, but the estimate was imprecise and the confidence interval included the null because only three trials were available.
Definition of a ketogenic diet	A ketogenic diet was defined as providing <50 g carbohydrate/day or <10% of total energy intake from carbohydrate.	A ketogenic diet was operationally defined as providing ≤50 g carbohydrate/day or ≤10% of total energy intake from carbohydrate. When both prescribed and achieved carbohydrate intake were reported, achieved intake was used to determine eligibility.	The boundary was clarified to allow consistent classification of interventions reported exactly at the prespecified threshold. Prioritizing achieved intake was intended to reflect the dietary exposure actually attained during the intervention rather than the prescribed target alone.	The clarification reduced the risk of misclassifying interventions on the basis of their label or prescribed composition. No trial was included solely because of changing the boundary from < to ≤; prioritizing achieved intake affected the assessment of interventions in which the attained carbohydrate intake did not meet the ketogenic threshold.

Search completion date	The literature search was planned to be completed before 31 August 2025.	Database searches were conducted on 9 October 2025 and 14 October 2025.	The searches were conducted after prospective registration and were completed before study selection to capture evidence available at the time the review commenced.	The later search dates increased the currency of the evidence base without narrowing the registered search scope or changing the eligibility criteria.
Subgroup analysis by intervention duration	A subgroup analysis comparing interventions lasting ≤6 months with those lasting >6 months was planned.	The subgroup analysis was not performed.	Only one trial had an intervention duration >6 months; consequently, a formal subgroup comparison would have been statistically uninformative and potentially misleading.	The possibility of effect modification by intervention duration could not be assessed quantitatively and was considered narratively when interpreting the findings.

PROSPERO, International Prospective Register of Systematic Reviews.

Supplementary Table S3. Full Electronic Search Strategies

Database	Variant	Full String (exactly as run)	Filters in GUI	Date Searched (YYYY-MM-DD)	Records Retrieved (n)
PubMed (MEDLINE)	A (broad)	((“Obesity”[MeSH Terms] OR “Overweight”[MeSH Terms] OR (“obeses”[All Fields] OR “Obesity”[MeSH Terms] OR “Obesity”[All Fields] OR “obese”[All Fields] OR “obesities”[All Fields] OR “obesity s”[All Fields]) OR (“Overweight”[MeSH Terms] OR “Overweight”[All Fields] OR “overweighted”[All Fields] OR “overweightness”[All Fields] OR “overweights”[All Fields]) OR “body mass index”[All Fields] OR “BMI”[All Fields]) AND	English; Article; Randomized Controlled Trial / Clinical Trial; All years	2025-10-09	294

		(“diet, ketogenic”[MeSH Terms] OR “ketogenic diet”[All Fields] OR “very low carbohydrate diet”[All Fields] OR “low carbohydrate diet”[All Fields] OR “vlcd”[All Fields] OR “keto diet”[All Fields])) AND ((randomizedcontrolledtrial[Filter]) AND (humans[Filter]) AND (english[Filter]) AND (alladult[Filter]))			
PubMed (MEDLINE)	B (specific: fat loss/body composition)	((“Obesity”[Mesh] OR “Overweight”[Mesh] OR obesity OR overweight OR “body mass index” OR BMI) AND (“Diet, Ketogenic”[Mesh] OR “ketogenic diet” OR “very low carbohydrate diet” OR “low carbohydrate diet” OR VLCKD OR “keto diet”) AND (“body composition” OR “fat mass” OR “body fat” OR “fat loss” OR “weight loss” OR “weight change” OR “weight reduction”))	English; Article; Randomized Controlled Trial / Clinical Trial; All years	2025-10-09	174
Scopus	A (broad)	TITLE-ABS-KEY ((obesity OR overweight OR “body mass index” OR BMI) AND (“ketogenic diet” OR “very low carbohydrate diet” OR “low carbohydrate diet” OR VLCKD OR “keto diet”) AND (“randomized controlled trial” OR “randomised controlled trial” OR randomized OR randomised OR “randomly assigned” OR “controlled trial” OR “intervention study”))	-	2025-10-09	1200
Scopus	B (specific: fat loss/body composition)	TITLE-ABS-KEY ((obesity OR overweight OR “body mass index” OR BMI) AND (“ketogenic diet” OR “very low carbohydrate diet” OR	-	2025-10-09	888

		"low carbohydrate diet" OR VLCKD OR "keto diet") AND ("body composition" OR "fat mass" OR "body fat" OR "fat loss" OR "weight loss" OR "weight change" OR "weight reduction") AND ("randomized controlled trial" OR "randomised controlled trial" OR randomized OR randomised OR "randomly assigned" OR "controlled trial" OR "intervention study"))			
Web of Science Core Collection	A (broad)	TS=((obesity OR overweight OR "body mass index" OR BMI) AND ("ketogenic diet" OR "very low carbohydrate diet" OR "low carbohydrate diet" OR VLCKD OR "keto diet") AND ("randomized controlled trial" OR "randomised controlled trial" OR randomized OR randomised OR "randomly assigned" OR "controlled trial" OR "intervention study"))	-	2025-10-14	758
Web of Science Core Collection	B (specific: fat loss/body compositi on)	TS=((obesity OR overweight OR "body mass index" OR BMI) AND ("ketogenic diet" OR "very low carbohydrate diet" OR "low carbohydrate diet" OR VLCKD OR "keto diet") AND ("body composition" OR "fat mass" OR "body fat" OR "fat loss" OR "weight loss" OR "weight change" OR "weight reduction") AND ("randomized controlled trial" OR "randomised controlled trial" OR randomized OR randomised OR "randomly assigned" OR "controlled trial" OR "intervention study"))	-	2025-10-14	645

Supplementary Table S4. Reports Excluded After Full-Text Assessment and Reasons for Exclusion

First author, year	Full citation	Primary reason for exclusion	Standardized exclusion category
Abete 2009	Abete I et al. Effects of two energy-restricted diets differing in the carbohydrate/protein ratio on weight loss and oxidative changes of obese men. International Journal of Food Sciences and Nutrition. 2009.	Intervention not ketogenic or very low-carbohydrate as specified in the protocol	Ineligible intervention
Abu Salma 2025	Abu Salma BM et al. Papel de la dieta cetogénica modificada en la pérdida de peso y los parámetros metabólicos. Revista Española de Nutrición Comunitaria. 2025.	No eligible comparator group	Ineligible comparator
Alexandrakis 2015	Alexandrakis I, Palacio C, Mooradian AD. Relative merits of low-carbohydrate versus low-fat diet in managing obesity. Southern Medical Journal. 2015;108(7):401–416.	Study is a review article, not a primary randomized controlled trial	Ineligible study design or publication type
Alkhorayef 2023	Alkhorayef N et al. Regulatory effects of ketogenic diet on the inflammatory response in obese Saudi women. Journal of Taibah University Medical Sciences. 2023;18(5):1101–1107.	No eligible comparator group	Ineligible comparator
Aller 2019	Aller R, Izaola O, Primo D, de Luis DA. The effect of single-nucleotide polymorphisms at the ADIPOQ gene locus rs1501299 on metabolic parameters after 9 mo of a high-protein/low-carbohydrate versus a standard hypocaloric diet. Nutrition. 2019;65:44–49.	Intervention not ketogenic or very low-carbohydrate as specified in the protocol	Ineligible intervention
Alsharif 2025	Alsharif DJ. Low-fat versus low-carbohydrate diet. Journal of Nature and Science of Medicine. 2025;8(1):1–5.	Review article without original randomized controlled trial data	Ineligible study design or publication type
Arai 1992	Arai K, Miura J, Ohno M, Yokoyama J, Ikeda Y. Comparison of clinical usefulness of very-low-calorie diet and supplemental low-calorie diet. American Journal of Clinical Nutrition. 1992;56:275S–276S.	No eligible comparator group	Ineligible comparator
Aronica 2021	Aronica L et al. Examining differences between overweight women and men in 12-month weight loss study comparing healthy low-carbohydrate vs healthy low-fat diets. International Journal of Obesity. 2021;45:225–234.	Intervention not ketogenic or very low-carbohydrate as specified in the protocol	Ineligible intervention
Basciani 2020	Basciani S et al. Very-Low-Calorie Ketogenic Diets With Whey, Vegetable, or Animal Protein in Patients With Obesity: A Randomized Pilot Study. Journal of Clinical Endocrinology & Metabolism. 2020;105(9):2939–2949.	No eligible comparator group	Ineligible comparator

Bazzano 2014	Bazzano LA et al. Effects of Low-Carbohydrate and Low-Fat Diets. Annals of Internal Medicine. 2014;161:309–318.	Intervention not ketogenic or very low-carbohydrate as specified in the protocol	Ineligible intervention
Bradley 2009	Bradley U et al. Low-Fat Versus Low-Carbohydrate Weight Reduction Diets: Effects on Weight Loss, Insulin Resistance, and Cardiovascular Risk. Diabetes. 2009;58:2741–2748.	Intervention not ketogenic or very low-carbohydrate as specified in the protocol	Ineligible intervention
Brehm 2003	Brehm BJ et al. A Randomized Trial Comparing a Very Low Carbohydrate Diet and a Calorie-Restricted Low-Fat Diet in Healthy Women. J Clin Endocrinol Metab. 2003;88:1617–1623.	Interventions not designed to be isocaloric	Energy-matching criterion not met
Brehm 2005	Brehm BJ et al. The Role of Energy Expenditure in the Differential Weight Loss in Obese Women on Low-Fat and Low-Carbohydrate Diets. J Clin Endocrinol Metab. 2005;90:1475–1482.	Interventions not designed to be isocaloric	Energy-matching criterion not met
Brinkworth 2004	Brinkworth GD, Noakes M, Keogh JB, Luscombe ND, Wittert GA, Clifton PM. Long-term effects of a high-protein, low-carbohydrate diet on weight control and cardiovascular risk markers in obese hyperinsulinemic subjects. International Journal of Obesity. 2004;28:661–670.	Intervention not ketogenic or very low-carbohydrate as specified in the protocol	Ineligible intervention
Brinkworth 2009	Brinkworth GD et al. Comparative effects of very low-carbohydrate, high-fat and high-carbohydrate, low-fat weight-loss diets on bowel habit and faecal short-chain fatty acids and bacterial populations. Br J Nutr. 2009;101:1493–1502.	Companion report of an included trial; no additional independent body weight data.	Duplicate or secondary report
Brinkworth 2010	Brinkworth GD et al. Renal Function Following Long-Term Weight Loss in Individuals with Abdominal Obesity on a Very-Low-Carbohydrate Diet vs High-Carbohydrate Diet. J Am Diet Assoc. 2010;110:633–638.	Duplicate or overlapping cohort	Duplicate or secondary report
Brinkworth 2016	Brinkworth GD et al. Long-term effects of very low-carbohydrate and high-carbohydrate weight-loss diets on psychological health in obese adults with type 2 diabetes. J Intern Med. 2016.	Companion report of an included trial; no additional independent body weight data.	Duplicate or secondary report
Brinkworth 2016	Brinkworth GD et al. Long-term effects of a very-low-carbohydrate weight-loss diet and an isocaloric low-fat diet on bone health in obese adults. Nutrition. 2016.	Companion report of an included trial; no additional independent body weight data.	Duplicate or secondary report
Buga 2021	Buga A et al. The Effects of a 6-Week Controlled, Hypocaloric Ketogenic Diet, With and Without Exogenous Ketone Salts, on Body	Comparator group not randomized within the trial	Ineligible study design

	Composition Responses. Frontiers in Nutrition. 2021;8:618520.		or publication type
Buga 2023	Buga A et al. Fasting and diurnal blood ketonemia and glycemia responses to a six-week, energy-controlled ketogenic diet supplemented with ketone salts. Clin Nutr ESPEN. 2023;54:277–287.	Secondary publication from an already assessed cohort	Duplicate or secondary report
Burgess 2017	Burgess B et al. PROP Nontaster Women Lose More Weight Following a Low-Carbohydrate Versus a Low-Fat Diet in a Randomized Controlled Trial. Obesity. 2017;25:1682–1690.	Non-isocaloric dietary intervention	Energy-matching criterion not met
Buscemi 2009	Buscemi S et al. Effects of hypocaloric very-low-carbohydrate diet vs. Mediterranean diet on endothelial function in obese women. Eur J Clin Invest. 2009;39(5):339–347.	Intervention not ketogenic or very low-carbohydrate as specified in the protocol	Ineligible intervention
Cai 2021	Cai L et al. Low-carbohydrate diets lead to greater weight loss and better glucose homeostasis than exercise: a randomized clinical trial. Front Med. 2021;15(3):460–471.	Comparator not eligible (no higher-carbohydrate diet control group)	Ineligible comparator
Cano-Rodríguez 2006	Cano-Rodríguez I et al. Dietas bajas en hidratos de carbono frente a dietas bajas en grasas. Endocrinol Nutr. 2006;53(3):209–217.	Review article, no original randomized controlled trial data	Ineligible study design or publication type
Caprio 2023	Caprio M et al. Very low-calorie ketogenic diet versus hypocaloric balanced diet in migraine prevention: a randomized controlled trial. Journal of Translational Medicine. 2023;21:561.	Non-isocaloric dietary intervention	Energy-matching criterion not met
Castellana 2022	Castellana M, Conte E, Cignarelli A, Perrini S, Giustina A, Giorgino F. Efficacy and safety of very-low-calorie ketogenic diets in subjects with obesity: a randomized controlled trial. Endocrine Connections. 2022;11:e220536.	Intervention not isocaloric as specified in the protocol	Energy-matching criterion not met
Christensen 2021	Christensen RAG et al. Sequential diets and weight loss: Including a low-carbohydrate high-fat diet with and without time-restricted feeding. Nutrition. 2021;91–92:111393.	Study design not eligible (non-randomized sequential case-crossover without parallel comparator groups)	Ineligible study design or publication type
Cipryan 2020	Cipryan L et al. Effects of a four-week very low-carbohydrate high-fat diet on biomarkers of inflammation: Non-randomised parallel-group study. Nutrition and Health. 2020;26:1–8.	Non-randomized study design	Ineligible study design or publication type
Colica 2017	Colica C et al. Efficacy and safety of very-low-calorie ketogenic diet: a double-blind randomized crossover study. Eur Rev Med Pharmacol Sci. 2017;21:2274–2289.	Comparator not eligible (no higher-carbohydrate diet control group)	Ineligible comparator

Crabtree 2021	Crabtree CD et al. Comparison of Ketogenic Diets with and without Ketone Salts versus a Low-Fat Diet: Liver Fat Responses in Overweight Adults. Nutrients. 2021;13:966.	Comparator group not randomized within the trial	Ineligible study design or publication type
Cunha 2020	Cunha GM et al. Efficacy of a 2-Month Very Low-Calorie Ketogenic Diet (VLCKD) Compared to a Standard Low-Calorie Diet in Reducing Visceral and Liver Fat Accumulation in Patients With Obesity. Front Endocrinol. 2020;11:607.	Intervention not isocaloric	Energy-matching criterion not met
Currenti 2024	Currenti W et al. Comparative Evaluation of a Low-Carbohydrate Diet and a Mediterranean Diet in Overweight/Obese Patients with Type 2 Diabetes Mellitus: A 16-Week Intervention Study. Nutrients. 2024;16:95.	Non-randomized study	Ineligible study design or publication type
da Silva Schmitt 2023	da Silva Schmitt C et al. The effects of a low carbohydrate diet on erectile function and serum testosterone levels in hypogonadal men with metabolic syndrome: a randomized clinical trial. BMC Endocrine Disorders. 2023;23:30.	Intervention not ketogenic or very low-carbohydrate as specified in the protocol	Ineligible intervention
Davis 2009	Davis NJ et al. Comparative study of the effects of a 1-year dietary intervention of a low-carbohydrate diet versus a low-fat diet on weight and glycemic control in type 2 diabetes. Diabetes Care. 2009;32:1147–1152.	Intervention not ketogenic or very low-carbohydrate as specified in the protocol	Ineligible intervention
de Luis 2007	de Luis DA et al. Effects of a Low-Fat versus a Low-Carbohydrate Diet on Adipocytokines in Obese Adults. Horm Res. 2007;67:296–300.	Intervention not ketogenic or very low-carbohydrate as specified in the protocol	Ineligible intervention
de Luis 2008	de Luis DA et al. Influence of Lys656Asn polymorphism of leptin receptor gene on leptin response secondary to two hypocaloric diets: a randomized clinical trial. Ann Nutr Metab. 2008;52:209–214.	Intervention not ketogenic or very low-carbohydrate as specified in the protocol	Ineligible intervention
de Luis 2008	Influence of Ala54Thr polymorphism of fatty acid-binding protein 2 on weight loss and insulin levels secondary to two hypocaloric diets. Diabetes Research and Clinical Practice. 2008	Intervention not ketogenic diet	Ineligible intervention
de Luis 2009	Influence of Trp64Arg polymorphism of beta 3-adrenoreceptor gene on insulin resistance, adipocytokines and weight loss secondary to two hypocaloric diets. Ann Nutr Metab. 2009;54:104–110	Intervention not ketogenic or very low-carbohydrate as specified in the protocol	Ineligible intervention
de Luis 2009	Efectos de una dieta baja en grasas frente a una dieta rica en proteínas y grasa en pacientes obesos. Med Clin (Barc). 2009;132(6):203–207	Intervention not ketogenic or very low-carbohydrate as specified in the protocol	Ineligible intervention

de Luis 2012	de Luis DA et al. Role of G1359A polymorphism of the cannabinoid receptor gene on weight loss and adipocytokines levels after two different hypocaloric diets. Journal of Nutritional Biochemistry. 2012;23:287–291.	Intervention not ketogenic or very low-carbohydrate as specified in the protocol	Ineligible intervention
de Luis 2015	de Luis DA et al. Effects of a High Protein/Low Carbohydrate versus a Standard Hypocaloric Diet on Adipocytokine Levels and Insulin Resistance in Obese Patients along 9 Months. J Diabetes Complications. 2015	Intervention not ketogenic / carbohydrate intake >50 g/day	Ineligible intervention
de Luis 2020	de Luis DA et al. A circadian rhythm-related MTNR1B genetic variant (rs10830963) modulate body weight change and insulin resistance after 9 months of a high protein/low carbohydrate vs a standard hypocaloric diet Journal of Diabetes and Its Complications 2020;34:107534	Non-ketogenic low-carbohydrate intervention	Ineligible intervention
de Luis 2021	de Luis DA, Primo Martín D, Izaola O. Adiponectin gene variant rs266729 interacts with different macronutrient distributions of two different hypocaloric diets during nine months Nutr Hosp 2021;38(2):274–280	Non-ketogenic low-carbohydrate intervention	Ineligible intervention
De Natale 2009	De Natale C et al. Effects of a Plant-Based High-Carbohydrate/High-Fiber Diet Versus High–Monounsaturated Fat/Low-Carbohydrate Diet on Postprandial Lipids in Type 2 Diabetic Patients. Diabetes Care. 2009	Not a ketogenic or very-low-carbohydrate intervention	Ineligible intervention
Dening 2023	Dening J et al. A web-based low carbohydrate diet intervention significantly improves glycaemic control in adults with type 2 diabetes: results of the T2Diet Study randomised controlled trial. Nutr Diabetes. 2023;13:12	Intervention not ketogenic or very low-carbohydrate as specified in the protocol	Ineligible intervention
Di Lorenzo 2019	Di Lorenzo C et al. A randomized double-blind, cross-over trial of very low-calorie diet in overweight migraine patients: A possible role for ketones? Nutrients. 2019;11:1742	Intervention not isocaloric as specified in the protocol	Energy-matching criterion not met
Doola 2019	Doola R et al. The effect of a low carbohydrate formula on glycaemia in critically ill enterally-fed adult patients with hyperglycaemia: A blinded randomised feasibility trial. Clin Nutr ESPEN. 2019	The study population consisted of critically ill, enterally fed adults and did not meet the predefined population criteria.	Ineligible population
Dou 2024	Dou P et al. A Randomized Trial of the Efficacy of Three Weight Loss Diet Interventions in Overweight/Obese with Polycystic Ovary Syndrome. Endocr Metab Immune Disord Drug Targets. 2024	Intervention not ketogenic or very low-carbohydrate as specified in the protocol	Ineligible intervention

Drabińska 2023	Drabińska N et al. The effect of isocaloric, energy-restrictive ketogenic diet on metabolism, inflammation, nutrition deficiencies and oxidative stress in women with overweight and obesity (KETO-MINOX): Study protocol. PLoS ONE. 2023	Study protocol without outcome data	Ineligible study design or publication type
Du 2025	Du Y et al. Digitally enhanced ketogenic diet versus low-fat diet for cardio-renal-metabolic health. Diabetes Obes Metab. 2025.	The compared dietary interventions were not prescribed with the same energy intake or an equivalent target energy deficit.	Energy-matching criterion not met
Dyson 2007	Dyson PA, Beatty S, Matthews DR. A low-carbohydrate diet is more effective in reducing body weight than healthy eating in both diabetic and non-diabetic subjects. Diabet Med. 2007;24:1430–1435	Intervention not isocaloric as specified in the protocol	Energy-matching criterion not met
Ebbeling 2007	Ebbeling CB et al. Effects of a Low–Glycemic Load vs Low-Fat Diet in Obese Young Adults: A Randomized Trial. JAMA. 2007	Intervention not ketogenic or very low-carbohydrate as specified in the protocol	Ineligible intervention
Ebbeling 2022	Ebbeling CB et al. Effects of a low-carbohydrate diet on insulin-resistant dyslipoproteinemia—a randomized controlled feeding trial. Am J Clin Nutr. 2022	Intervention not ketogenic or very low-carbohydrate as specified in the protocol	Ineligible intervention
Elhayany 2010	Elhayany A et al. A low carbohydrate Mediterranean diet improves cardiovascular risk factors and diabetes control among overweight patients with type 2 diabetes mellitus: a 1-year prospective randomized intervention study. Diabetes Obes Metab. 2010;12:204–209.	Not ketogenic intervention (carbohydrate intake >50 g/day; not VLCKD)	Ineligible intervention
Elsahoryi 2023	Elsahoryi NA et al. Effects of a low-calorie ketogenic diet versus a low-carbohydrate diet on cardiometabolic outcomes in adults with obesity.	Comparator diet not aligned with protocol (no low-fat or high-carbohydrate control)	Ineligible comparator
Forsythe 2008	Forsythe CE et al. Comparison of low-carbohydrate and low-fat diets on circulating fatty acid composition and markers of inflammation. Lipids. 2008.	Diets were provided ad libitum without a prespecified energy-matched intervention plan.	Energy-matching criterion not met
Foster 2003	Foster GD et al. A Randomized Trial of a Low-Carbohydrate Diet for Obesity. N Engl J Med. 2003;348:2082–2090	Intervention not isocaloric as	Energy-matching

		specified in the protocol	criterion not met
Foster 2010	Foster GD et al. Weight and Metabolic Outcomes after 2 Years on a Low-Carbohydrate versus Low-Fat Diet: A Randomized Trial. N Engl J Med. 2010	Intervention not isocaloric as specified in the protocol	Energy-matching criterion not met
Frisch 2009	Frisch S et al. A randomized controlled trial on the efficacy of carbohydrate-reduced or fat-reduced diets in patients attending a telemedically guided weight loss program. Cardiovasc Diabetol. 2009;8:36	Intervention not ketogenic or very low-carbohydrate as specified in the protocol	Ineligible intervention
Gardner 2015	Gardner CD et al. Weight loss on low-fat vs. low-carbohydrate diets by insulin resistance status among overweight adults and adults with obesity: A randomized pilot trial. Obesity. 2016;24:79–86	Intervention not ketogenic or very low-carbohydrate as specified in the protocol	Ineligible intervention
Gardner 2018	Gardner CD et al. Effect of Low-Fat vs Low-Carbohydrate Diet on 12-Month Weight Loss in Overweight Adults. JAMA. 2018	Intervention not ketogenic or very low-carbohydrate as specified in the protocol	Ineligible intervention
Gepner 2019	Gepner Y, Shelef I, Komy O, et al. The beneficial effects of Mediterranean diet over low-fat diet may be mediated by decreasing hepatic fat content. Journal of Hepatology. 2019;71:379–388.	Secondary analysis of previously published cohort	Duplicate or secondary report
Goday 2016	Goday A. et al. Very low-calorie-ketogenic diet safety in T2DM. Nutrition & Diabetes. 2016;6:e230	Non-isocaloric intervention	Energy-matching criterion not met
Golay 1996	Golay A et al. Weight-loss with low or high carbohydrate diet? Int J Obes Relat Metab Disord. 1996	Intervention not ketogenic or very low-carbohydrate as specified in the protocol	Ineligible intervention
Goldstein 2011	Goldstein T et al. The effect of a low carbohydrate energy-unrestricted diet on weight loss in obese type 2 diabetes patients: a randomized controlled trial. e-SPEN Eur e-J Clin Nutr Metab. 2011	Intervention not isocaloric as specified in the protocol	Energy-matching criterion not met
Goss 2020	Goss AM et al. Effects of weight loss during a very low-carbohydrate diet on specific adipose tissue depots and insulin sensitivity in older adults with obesity: a randomized clinical trial. Nutr Metab (Lond). 2020	Intervention not isocaloric as specified in the protocol	Energy-matching criterion not met
Gram-Kampmann 2022	Gram-Kampmann EM et al. Effects of a 6-month, low-carbohydrate diet on glycaemic control, body composition, and cardiovascular risk factors in patients with type 2 diabetes: An open-label randomized controlled trial. Diabetes Obes Metab. 2022	Intervention not ketogenic or very low-carbohydrate as specified in the protocol	Ineligible intervention

Greenberg 2009	Greenberg I et al. Adherence and Success in Long-Term Weight Loss Diets: The Dietary Intervention Randomized Controlled Trial (DIRECT). J Am Coll Nutr. 2009	Secondary analysis of previously published cohort	Duplicate or secondary report
Grembi 2020	Grembi JA et al. Gut microbiota plasticity is correlated with sustained weight loss on a low-carb or low-fat dietary intervention. Sci Rep. 2020	Secondary analysis of previously published cohort	Duplicate or secondary report
Guldbrand 2012	Guldbrand H et al. Diabetologia. 2012;55:2118–2127.	Not ketogenic intervention (carbohydrate intake >50 g/day)	Ineligible intervention
Guldbrand 2014	Guldbrand H et al. Randomization to a low-carbohydrate diet advice improves health related quality of life compared with a low-fat diet at similar weight-loss in Type 2 diabetes mellitus. Diabetes Res Clin Pract. 2014	Secondary analysis of previously published cohort	Duplicate or secondary report
Guo 2022	Guo H et al. Effects of low-carbohydrate vs low-fat diets on weight loss and metabolic risk factors in obese/overweight individuals with impaired glucose regulation: A randomized controlled trial. Asia Pac J Clin Nutr. 2022	Intervention not ketogenic or very low-carbohydrate as specified in the protocol	Ineligible intervention
Haufe 2011	Haufe S et al. Randomized comparison of reduced fat and reduced carbohydrate hypocaloric diets on intrahepatic fat in overweight and obese human subjects. Hepatology. 2011	Not ketogenic intervention (carbohydrate intake >50 g/day)	Ineligible intervention
Haywood 2017	Haywood CJ et al. Very Low Calorie Diets for Weight Loss in Obese Older Adults—A Randomized Trial. J Gerontol A Biol Sci Med Sci. 2017	Non-ketogenic dietary intervention	Ineligible intervention
Heggen 2015	Heggen E et al. Effect of low-carbohydrate versus low-fat diet on weight change during smoking cessation: a randomized controlled trial. Nicotine Tob Res. 2015	The low-carbohydrate intervention did not meet the predefined ketogenic or very-low-carbohydrate criterion.	Ineligible intervention
Howard 2006	Howard BV et al. Low-fat dietary pattern and weight change over 7 years: the Women's Health Initiative Dietary Modification Trial. JAMA. 2006	Not a direct comparison of low-carbohydrate versus higher-carbohydrate diets	Ineligible comparator
Hu 2015	Hu T, Yao L, Reynolds K, Whelton PK, Niu T, Li S, He J, Bazzano LA. The Effects of a Low-Carbohydrate Diet vs. a Low-Fat Diet on Novel Cardiovascular Risk Factors: A Randomized Controlled Trial. Nutrients. 2015;7:7978–7994.	Intervention not isocaloric as specified in the protocol	Energy-matching criterion not met
Hwang 2020	Hwang CL, Ranieri C, Szczurek MR, Ellythy AM, Elokda A, Mahmoud AM, Phillips SA. The Effect of Low-Carbohydrate Diet on	No eligible higher-carbohydrate comparator as	Ineligible comparator

	Macrovascular and Microvascular Endothelial Function is Not Affected by the Provision of Caloric Restriction in Women with Obesity: A Randomized Study. Nutrients. 2020;12:1649.	specified in the protocol	
Iqbal 2010	Iqbal N, Vetter ML, Moore RH, Chittams JL, Dalton-Bakes CV, Dowd M, Williams-Smith C, Cardillo S, Wadden TA. Effects of a Low-intensity Intervention That Prescribed a Low-carbohydrate vs. a Low-fat Diet in Obese, Diabetic Participants. Obesity. 2010;18:1733–1738.	Intervention not isocaloric as specified in the protocol	Energy-matching criterion not met
Ismael 2021	Ismael SA. Effects of low carbohydrate diet compared to low fat diet on reversing the metabolic syndrome, using NCEP ATP III criteria: a randomized clinical trial. BMC Nutrition. 2021;7:62.	Study design not eligible	Ineligible study design or publication type
Izaola 2019	Izaola O, Primo D, Gómez Hoyos E, López Gómez JJ, Ortolá A, de Luis DA. Association of rs670 variant of APOA1 gene with lipid profile and insulin resistance after 9 months of a high protein/low carbohydrate vs a standard hypocaloric diet. Clinical Nutrition. 2020;39:988–993.	Intervention not ketogenic or very low-carbohydrate as specified in the protocol	Ineligible intervention
Jansen 2022	Jansen LT et al. Prolonged glycemic adaptation following transition from a low- to high-carbohydrate diet: a randomized controlled feeding trial. Diabetes Care. 2022.	The randomized comparison assessed glycaemic adaptation after a preceding weight-loss phase and did not provide an eligible comparison of body-weight change during the dietary intervention.	Ineligible outcome data
Jenkins 2009	Jenkins DJA, Wong JMW, Kendall CWC, Esfahani A, Ng VWY, Leong TCK, Faulkner DA, Vidgen E, Greaves KA, Paul G, Singer W. The Effect of a Plant-Based Low-Carbohydrate ("Eco-Atkins") Diet on Body Weight and Blood Lipid Concentrations in Hyperlipidemic Subjects. Arch Intern Med. 2009;169:1046–1054.	Intervention not ketogenic or very low-carbohydrate as specified in the protocol	Ineligible intervention
Jenkins 2014	Jenkins DJA et al. Effect of a 6-month vegan low-carbohydrate ('Eco-Atkins') diet on cardiovascular risk factors and body weight in hyperlipidaemic adults: a randomised controlled trial. BMJ Open. 2014;4:e003505.	Intervention not ketogenic or very low-carbohydrate as specified in the protocol	Ineligible intervention
Kackley 2024	Kackley ML et al. Self-reported menses physiology is positively modulated by a well-formulated, energy-controlled ketogenic diet	Comparison between the ketogenic and low-	Ineligible study design or publication type

	vs. low-fat diet in women with overweight/obesity. PLoS ONE. 2024.	fat diet groups was not randomized.	
Kakoschke 2021	Kakoschke N et al. Effects of very low-carbohydrate vs high-carbohydrate weight loss diets on psychological health in adults with obesity and type 2 diabetes: a 2-year randomized controlled trial. Eur J Nutr. 2021.	Linked report from a trial in which achieved carbohydrate intake exceeded the predefined ketogenic threshold.	Ineligible intervention
Kani 2017	Kani AH, Alavian SM, Esmaillzadeh A, Adibi P, Haghighatdoost F, Azadbakht L. Effects of a Low-Calorie, Low-Carbohydrate Soy Containing Diet on Systemic Inflammation Among Patients with Nonalcoholic Fatty Liver Disease: A Parallel Randomized Clinical Trial. Horm Metab Res. 2017;49:687–694.	Intervention not ketogenic or very low-carbohydrate as specified in the protocol	Ineligible intervention
Karbalaiee 2022	Karbalaiee M, Chiti H, Mousavi SN, Afshar D. Low-carbohydrate hypo calorie diet has a beneficial effect on gut phyla and metabolic markers in healthy women with obesity: A randomized crossover study. Obesity Medicine. 2022;35:100461.	Intervention not ketogenic or very low-carbohydrate as specified in the protocol	Ineligible intervention
Katsuya 2023	Katsuya S, Kawata Y, Goto T, Tsubota J. Daily Intake of d-β-Hydroxybutyric Acid (d-BHB) Reduces Body Fat in Japanese Adult Participants: A Randomized, Double-Blind, Placebo-Controlled Study. J Nutr Sci Vitaminol. 2023;69:121–128.	Intervention not a dietary ketogenic intervention as specified in the protocol	Ineligible intervention
Keogh 2008	Keogh JB et al. Effects of weight loss from a very-low-carbohydrate diet on endothelial function and markers of cardiovascular disease risk in subjects with abdominal obesity. Am J Clin Nutr. 2008.	Companion report of an included trial; no additional independent body weight data.	Duplicate or secondary report
Kikuchi 2023	Kikuchi T, Kushiyama A, Yanai M, Kashiwado C, Seto T, Kasuga M. Comparison of Weight Reduction, Change in Parameters and Safety of a Very Low Carbohydrate Diet in Comparison to a Low Carbohydrate Diet in Obese Japanese Subjects with Metabolic Disorders. Nutrients. 2023;15:1342.	No eligible higher-carbohydrate comparator as specified in the protocol	Ineligible comparator
Klein 2025	Klein J, Schmid A, Keller U, et al. Ketogenic Diets and Metabolic Health: Molecular Mechanisms and Clinical Implications. International Journal of Molecular Sciences. 2025;25:2475.	Review article without original randomized controlled trial data	Ineligible study design or publication type
Krauss 2006	Krauss RM, Blanche PJ, Rawlings RS, Fernstrom HS, Williams PT. Separate effects of reduced carbohydrate intake and weight loss on atherogenic dyslipidemia. Am J Clin Nutr. 2006;83:1025–1031.	Intervention not ketogenic or very low-carbohydrate as specified in the protocol	Ineligible intervention

Kreider 2011	Kreider RB, Rasmussen C, Kerksick CM, Wilborn C, Taylor L, Campbell B, Magrans-Courtney T, Fogt D, Ferreira M, Li R, Galbreath M, Iosia M, Cooke M, Serra M, Gutierrez J, Byrd M, Kresta JY, Simbo S, Oliver J, Greenwood M. A Carbohydrate-Restricted Diet During Resistance Training Promotes More Favorable Changes in Body Composition and Markers of Health in Obese Women With and Without Insulin Resistance. The Physician and Sportsmedicine. 2011;39:27–40.	Intervention not ketogenic or very low-carbohydrate as specified in the protocol	Ineligible intervention
Krejčí 2018	Krejčí M, Hainer V. Low-carbohydrate diets in the treatment of obesity. Vnitřní Lékařství. 2018;64:432–438.	Review article (non-English full text)	Ineligible study design or publication type
Lai 2025	Lai CQ, Parnell LD, Das SK, Gardner CD, Ordovás JM. Differential weight-loss responses of APOA2 genotype carriers to low-carbohydrate and low-fat diets: the DIETFITS trial. Obesity. 2025.	Secondary analysis of previously published cohort	Duplicate or secondary report
Landry 2021	Landry MJ, Crimarco A, Perelman D, et al. Adherence to Ketogenic and Mediterranean Study Diets in a Crossover Trial: The Keto–Med Randomized Trial. Nutrients. 2021;13:967.	Intervention not isocaloric as specified in the protocol	Energy-matching criterion not met
Larsen 2011	Larsen RN, Mann NJ, Maclean E, Shaw JE. The effect of high-protein, low-carbohydrate diets in the treatment of type 2 diabetes: a 12 month randomised controlled trial. Diabetologia. 2011;54:731–740.	Intervention not ketogenic or very low-carbohydrate as specified in the protocol	Ineligible intervention
LeCheminant 2007	LeCheminant JD, Gibson CA, Sullivan DK, Hall S, Washburn R, Vernon MC, Curry C, Stewart E, Westman EC, Donnelly JE. Comparison of a low carbohydrate and low fat diet for weight maintenance in overweight or obese adults enrolled in a clinical weight management program. Nutrition Journal. 2007;6:36.	Study design not eligible	Ineligible study design or publication type
Li 2021	Li J et al. Ketogenic diet in women with polycystic ovary syndrome and liver dysfunction who are obese: A randomized, open-label, parallel-group, controlled pilot trial. J Obstet Gynaecol Res. 2021.	Ineligible comparator	Ineligible comparator
Li 2024	Li X et al. Effects of healthy low-carbohydrate diet and time-restricted eating on body composition and metabolic health in adults with overweight or obesity: a randomized controlled trial. Cell Reports Medicine. 2024.	Intervention not ketogenic or very low-carbohydrate as specified in the protocol	Ineligible intervention
Lim 2010	Lim SS, Noakes M, Keogh JB, Clifton PM. Long-term effects of a low carbohydrate, low fat or high unsaturated fat diet compared to a	Ineligible comparator	Ineligible comparator

	no-intervention control. Nutrition, Metabolism & Cardiovascular Diseases. 2010;20:599–607.		
Lim 2024	Lim SL, Tay M, Ang SM, Wai SN, Ong KW, Neo WJ, Yap QV, Chan YH, Khoo CM. Development and Pragmatic Randomized Controlled Trial of Healthy Ketogenic Diet Versus Energy-Restricted Diet on Weight Loss in Adults with Obesity. Nutrients. 2024;16:4380.	Carbohydrate intake did not meet predefined ketogenic thresholds throughout the intervention	Ineligible intervention
Liu 2013	Liu X, Zhang G, Ye X, Li H, Chen X, Tang L, Feng Y, Shai I, Stampfer MJ, Hu FB, Lin X. Effects of a low-carbohydrate diet on weight loss and cardiometabolic profile in Chinese women: a randomised controlled feeding trial. British Journal of Nutrition. 2013;110:1444–1453.	Intervention not ketogenic or very low-carbohydrate as specified in the protocol	Ineligible intervention
Liu 2024	Liu Z, Jin P, Liu Y, Zhang Z, Wu X, Weng M, Cao S, Wang Y, Zeng C, Yang R, Liu C, Sun P, Tian C, Li N, Zeng Q. A comprehensive approach to lifestyle intervention based on a calorie-restricted diet ameliorates liver fat in overweight/obese patients with NAFLD: a multicenter randomized controlled trial in China. Nutrition Journal. 2024;23:64.	Intervention not ketogenic or very low-carbohydrate as specified in the protocol	Ineligible intervention
Llanos 2014	Llanos AA, Krok JL, Peng J, Pennell ML, Olivo-Marston S, Vitolins MZ, DeGraffinreid CR, Paskett ED. Favorable effects of low-fat and low-carbohydrate dietary patterns on serum leptin, but not adiponectin, among overweight and obese premenopausal women: a randomized trial. SpringerPlus. 2014;3:175.	Intervention not ketogenic or very low-carbohydrate as specified in the protocol	Ineligible intervention
Lobley 2014	Lobley GE et al. Glucose uptake by the brain on chronic high-protein weight-loss diets with either moderate or low amounts of carbohydrate. Br J Nutr. 2014.	Intervention duration did not exceed the predefined minimum of 4 weeks.	Ineligible intervention duration
Lockwood 2008	Lockwood CM, Moon JR, Tobkin SE, Walter AA, Smith AE, Dalbo VJ, Cramer JT, Stout JR. Minimal nutrition intervention with high-protein/low-carbohydrate and low-fat, nutrient-dense food supplement improves body composition and exercise benefits in overweight adults: a randomized controlled trial. Nutrition & Metabolism. 2008;5:11.	Intervention not ketogenic or very low-carbohydrate as specified in the protocol	Ineligible intervention
London 2024	London A et al. The impact of short-term eucaloric low- and high-carbohydrate diets on liver triacylglycerol content in males with overweight and obesity: a randomized crossover study. Am J Clin Nutr. 2024.	Intervention duration was shorter than the predefined minimum of more than 4 weeks.	Ineligible intervention duration

Lowndes 2012	Lowndes J, Kawiecki D, Pardo S, Nguyen V, Melanson KJ, Yu Z, Rippe JM. The effects of four hypocaloric diets containing different levels of sucrose or high fructose corn syrup on weight loss and related parameters. Nutrition Journal. 2012;11:55.	Intervention not ketogenic or very low-carbohydrate as specified in the protocol	Ineligible intervention
López-Moreno 2024	López-Moreno J et al. Effects of a ketogenic diet versus a standard low-fat diet on body weight and cardiometabolic risk factors: a randomized controlled trial. Nutr J. 2024.	Interventions not isocaloric as specified in the protocol	Energy-matching criterion not met
Lundanes 2024	Lundanes J, Gårseth M, Taylor S, Crescenzi R, Pridmore M, Wagnild R, Hyldmo ÅA, Martins C, Nymo S. The effect of a low-carbohydrate diet on subcutaneous adipose tissue in females with lipedema. Frontiers in Nutrition. 2024;11:1484612.	Population not eligible (lipedema-specific population)	Ineligible population
Luong 2024	Luong TV et al. A 3-Week Ketogenic Diet Increases Skeletal Muscle Insulin Sensitivity in Individuals With Obesity. Diabetes. 2024.	Short-term mechanistic crossover study	Ineligible intervention duration
Ma 2006	Ma Y, Olendzki BC, Hafner AR, Chiriboga DE, Culver AL, Andersen VA, Merriam PA, Pagoto SL. Low-carbohydrate and high-fat intake among adult patients with poorly controlled type 2 diabetes mellitus. Nutrition. 2006;22:1129–1136.	Study design not eligible	Ineligible study design or publication type
Martin 2011	Martin CK et al. Change in Food Cravings, Food Preferences, and Appetite During a Low-Carbohydrate and Low-Fat Diet. Obesity. 2011.	Low-carbohydrate intervention was prescribed ad libitum, whereas the comparator was calorie restricted.	Energy-matching criterion not met
Martínez-Montoro 2025	Martínez-Montoro JI et al. Effect of a ketogenic diet, time-restricted eating, or alternate-day fasting on weight loss in adults with obesity. BMC Medicine. 2025.	No eligible energy-matched higher-carbohydrate dietary comparator was included.	Ineligible comparator
Martins 2025	Martins C, Bryan DR, Sweatt SK, Garvey WT, Fontaine KR, Dutton GR, Gower BA. The impact of a low-carbohydrate (vs. low-fat) diet on fat mass loss in African American women is modulated by insulin sensitivity. Obesity. 2025;33:257–266.	Intervention not ketogenic or very low-carbohydrate as specified in the protocol	Ineligible intervention
McCarter 2003	McCarter DF. Low-carbohydrate diet effective for adults. Journal of Family Practice. 2003.	Article type not eligible (commentary / secondary summary)	Ineligible study design or publication type
McClernon 2007	McClernon FJ, Yancy WS Jr, Eberstein JA, Atkins RC, Westman EC. The effects of a low-carbohydrate ketogenic diet and a low-fat diet	Intervention not isocaloric as specified in the protocol	Energy-matching criterion not met

	on mood, hunger, and other self-reported symptoms. Obesity. 2007;15:182–187.		
McVay 2014	McVay MA, Voils CI, Coffman CJ, Geiselman PJ, Kolotkin RL, Mayer SB, Smith VA, Gaillard L, Turner MJ, Yancy WS Jr. Factors associated with choice of a low-fat or low-carbohydrate diet during a behavioral weight loss intervention. Appetite. 2014.	Study design not eligible	Ineligible study design or publication type
Mei 2022	Mei J, Li Y, Wang Q, Sun X, Zhang H, Wang H. Effects of a low-carbohydrate diet on body composition, metabolic profile and reproductive hormones in women with polycystic ovary syndrome: a randomized controlled trial. Frontiers in Nutrition. 2022;9:876620.	Intervention not ketogenic or very low-carbohydrate as specified in the protocol	Ineligible intervention
Merra 2016	Merra G, Miranda R, Barrucco S, Gualtieri P, Mazza M, Moriconi E, Marchese C, Chang TFM, De Lorenzo A, Di Renzo L. Very-low-calorie ketogenic diet with aminoacid supplement versus very low restricted-calorie diet for preserving muscle mass during weight loss: a pilot double-blind study. European Review for Medical and Pharmacological Sciences. 2016;20:2613–2621.	No eligible comparator group	Ineligible comparator
Merra 2017	Merra G, Miranda R, Barrucco S, Gualtieri P, Mazza M, Moriconi E, Marchese C, Chang TFM, De Lorenzo A, Di Renzo L. Effects of very-low-calorie diet on body composition, metabolic state, and genes expression. European Review for Medical and Pharmacological Sciences. 2017;21:329–345.	Intervention not isocaloric as specified in the protocol	Energy-matching criterion not met
Michalczyk 2020	Michalczyk MM et al. The Effects of a Low Calorie Ketogenic Diet on Glycaemic Control Variables in Hyperinsulinemic Overweight/Obese Females. Nutrients. 2020	Interventions not isocaloric as specified in the protocol	Energy-matching criterion not met
Michalopoulou 2024	Michalopoulou M, et al. REduced-Carbohydrate intervention for managing Obesity and Reduction of gestational Diabetes (RECORD): a randomized controlled feasibility trial. Diabetes, Obesity and Metabolism. 2024.	Population not eligible	Ineligible population
Mirza 2013	Mirza NM, et al. Effects of a low glycemic load or a low-fat dietary intervention on body weight in obese Hispanic American children and adolescents: a randomized controlled trial. Am J Clin Nutr. 2013.	Population not eligible	Ineligible population
Miyashita 2004	Miyashita Y, Koide N, Ohtsuka M, Ozaki H, Itoh Y, Oyama T, Uetake T, Ariga K, Shirai K. Beneficial effect of low carbohydrate in low calorie diets on visceral fat reduction in type 2 diabetic patients with obesity. Diabetes	Intervention not ketogenic or very low-carbohydrate as specified in the protocol	Ineligible intervention

	Research and Clinical Practice. 2004;65:235–241.		
Mogul 2014	Mogul HR, Freeman R, Nguyen K, Frey M, Klein LA, Jozak S, Tanenbaum K. Carbohydrate Modified Diet & Insulin Sensitizers Reduce Body Weight & Modulate Metabolic Syndrome Measures in EMPOWIR: A Randomized Trial of Normoglycemic Women with Midlife Weight Gain. PLoS ONE. 2014;9:e108264.	Intervention not ketogenic or very low-carbohydrate as specified in the protocol	Ineligible intervention
Mohler 2013	Mohler ER III, Sibley AA, Stein R, Davila-Roman V, Wyatt H, Badellino K, Rader DJ, Klein S, Foster GD. Endothelial Function and Weight Loss: Comparison of Low-Carbohydrate and Low-Fat Diets. Obesity. 2013;21:504–509.	Duplicate publication from the same cohort	Duplicate or secondary report
Moreno 2014	Moreno B, Bellido D, Sajoux I, Goday A, Saavedra D, Crujeiras AB, Casanueva FF. Comparison of a very low-calorie-ketogenic diet with a standard low-calorie diet in the treatment of obesity. Endocrine. 2014.	Intervention not isocaloric as specified in the protocol	Energy-matching criterion not met
Moreno 2016	Moreno B, Crujeiras AB, Bellido D, Sajoux I, Casanueva FF. Obesity treatment by very low-calorie-ketogenic diet at two years: reduction in visceral fat and on the burden of disease. Endocrine. 2016;54:681–690.	Intervention not isocaloric as specified in the protocol	Energy-matching criterion not met
Morris 2019	Morris E, Aveyard P, Dyson P, et al. A food-based, low-energy, low-carbohydrate diet for people with type 2 diabetes in primary care: A randomized controlled feasibility trial. Diabetes Obesity and Metabolism. 2020;22:512–520.	Intervention not isocaloric as specified in the protocol	Energy-matching criterion not met
Mousavi 2023	Mousavi SM, Ejtahed HS, Ettehad Marvasti F, Taghavi MS, Siadat SD, Hasani-Ranjbar S, Larijani B, Esmaillzadeh A. The effect of a moderately restricted carbohydrate diet on cardiometabolic risk factors in overweight and obese women with metabolic syndrome: a randomized controlled trial. Clinical Therapeutics. 2023;45:e103–e114.	Intervention not ketogenic or very low-carbohydrate as specified in the protocol	Ineligible intervention
Muzio 2007	Muzio F, Mondazzi L, Harris WS, Sommariva D, Branchi A. Effects of moderate variations in the macronutrient content of the diet on cardiovascular disease risk factors in obese patients with the metabolic syndrome. American Journal of Clinical Nutrition. 2007;86:946–951.	Intervention not ketogenic or very low-carbohydrate as specified in the protocol	Ineligible intervention
Nachon Garcia 2025	Nachon Garcia FJ et al. A randomized controlled trial comparing a very low-calorie low-fat ketogenic diet with a standard	Intervention not isocaloric as specified in the protocol	Energy-matching criterion not met

	hypocaloric diet in adults with class I obesity. Obesity Medicine. 2025;58:100642.		
Naude 2022	Naude CE, Brand A, Schoonees A, Nguyen KA, Chaplin M, Volmink J. Low-carbohydrate versus balanced-carbohydrate diets for reducing weight and cardiovascular risk. Cochrane Database of Systematic Reviews. 2022;Issue 1:CD013334. doi:10.1002/14651858.CD013334.pub2.	Study is a review article, not a primary randomized controlled trial	Ineligible study design or publication type
Nielsen 2006	Nielsen JV, Joensson E. Low-carbohydrate diet in type 2 diabetes. Stable improvement of bodyweight and glycemic control during 22 months follow-up. Nutrition & Metabolism. 2006;3:22.	Non-randomized study design	Ineligible study design or publication type
Niemczyk 2015	Niemczyk NA. Low-carbohydrate versus low-fat diet: A randomized clinical trial. Journal of Midwifery & Women's Health. 2015;60:104–106.	Study design not eligible	Ineligible study design or publication type
Noakes 2013	Noakes TD. Low-carbohydrate and high-fat intake can manage obesity and associated conditions: occasional survey. South African Medical Journal. 2013;103(11):826–830.	Study design not eligible	Ineligible study design or publication type
Padwal 2007	Padwal R. The Atkins diet led to more weight loss than the Zone diet in overweight and obese premenopausal women at 12 months. Evidence-Based Medicine. 2007;12:138.	Study design not eligible	Ineligible study design or publication type
Perissiou 2020	Perissiou M, Borkoles E, Kobayashi K, Polman R. The effect of an 8 week prescribed exercise and low-carbohydrate diet on cardiorespiratory fitness, body composition and cardiometabolic risk factors in obese individuals: a randomized controlled trial. Nutrients. 2020;12:482.	Intervention not isocaloric as specified in the protocol	Energy-matching criterion not met
Pescari 2025	Pescari D, Mihuta MS, Bena A, Stoian D. Comparative Effects of Time-Restricted Eating and the Ketogenic Diet on QRISK3-Assessed Cardiovascular Risk in Individuals with Obesity: A Longitudinal Analysis. Nutrients. 2025;17:1963.	Study design not eligible	Ineligible study design or publication type
Petersen 2006	Petersen M, Taylor MA, Saris WHM, et al. Randomized, multi-center trial of two hypo-energetic diets in obese subjects: high- versus low-fat content. International Journal of Obesity. 2006;30:552–560.	Intervention not ketogenic or very low-carbohydrate as specified in the protocol	Ineligible intervention
Petrisko 2020	Petrisko M, Kloss R, Bradley P, Birrenkott E, Spindler A, Clayton ZS, Kern M. Biochemical, anthropometric, and physiological responses to carbohydrate-restricted diets versus a low-fat diet in obese adults: a randomized crossover trial. Journal of Medicinal Food. 2020;23:1–9.	Intervention not isocaloric as specified in the protocol	Energy-matching criterion not met

Pinsawas 2024	Pinsawas S, et al. Asian low-carbohydrate diet with increased whole egg consumption improves metabolic outcomes in individuals with metabolic syndrome: a randomized controlled trial. Journal of Nutrition. 2024.	Intervention not isocaloric as specified in the protocol	Energy-matching criterion not met
Popp 2022	Popp CJ et al. Effect of a Personalized Diet to Reduce Postprandial Glycemic Response vs a Low-Fat Diet on Weight Loss in Adults With Abnormal Glucose Metabolism and Obesity. JAMA Network Open. 2022;5(9):e2233760.	Intervention not ketogenic or very low-carbohydrate as specified in the protocol	Ineligible intervention
Primo 2020	Primo D, Izaola O, de Luis D. Effects of a high protein/low carbohydrate low-calorie diet versus a standard low-calorie diet on anthropometric parameters and cardiovascular risk factors: role of polymorphism rs3123554 in the cannabinoid receptor gene type 2 (CB2R). Endocrinología, Diabetes y Nutrición. 2020;67:446–453.	Intervention not ketogenic or very low-carbohydrate as specified in the protocol	Ineligible intervention
Qi 2018	Qi L, Bray GA, Sacks FM. Low-Fat vs Low-Carbohydrate Diets and Weight Loss. Comment & Response. JAMA. 2018;320(2):202–203.	Study design not eligible	Ineligible study design or publication type
Rahman 2018	Rahman VJ. Low-Fat vs Low-Carbohydrate Diets and Weight Loss. Comment & Response. JAMA. 2018;320(2):202.	Study design not eligible	Ineligible study design or publication type
Rajaie 2013	Rajaie S, Azadbakht L, Saneei P, Khazaei M, Esmaillzadeh A. Comparative effects of carbohydrate versus fat restriction on serum levels of adipocytokines, markers of inflammation, and endothelial function among women with the metabolic syndrome: a randomized cross-over clinical trial. Annals of Nutrition and Metabolism. 2013;63:159–167.	Intervention not ketogenic or very low-carbohydrate as specified in the protocol	Ineligible intervention
Raygan 2016	Raygan F, Bahmani F, Kouchaki E, Aghadavod E, Sharifi S, Akbari E, Heidari A, Asemi Z. Comparative effects of carbohydrate versus fat restriction on metabolic profiles, biomarkers of inflammation and oxidative stress in overweight patients with type 2 diabetes mellitus and coronary heart disease: a randomized clinical trial. ARYA Atheroscler. 2016;12(6):266–273.	Intervention not ketogenic or very low-carbohydrate as specified in the protocol	Ineligible intervention
Rock 2014	Rock CL, Flatt SW, Sherwood NE, Karanja N, Pakiz B, Thomson CA. Effect of a free prepared meal and incentivized weight loss program on weight loss and metabolic outcomes in overweight and obese adults with type 2 diabetes: a randomized controlled trial. Obesity. 2014;22:1573–1581.	Comparator not eligible (multi-component commercial weight-loss program, not a diet-only comparison)	Ineligible comparator

Rock 2016	Rock CL, Flatt SW, Pakiz B, Quintana EL, Heath DD, Rana BK, Natarajan L. Effects of diet composition on weight loss, metabolic factors and biomarkers in a 1-year weight loss intervention in obese women examined by baseline insulin resistance status. Metabolism. 2016;65:1605–1613.	Intervention not ketogenic or very low-carbohydrate as specified in the protocol	Ineligible intervention
Röhling 2020	Röhling M, Martin K, Ellinger S, Schreiber M, Martin S, Kempf K. Weight Reduction by the Low-Insulin-Method—A Randomized Controlled Trial. Nutrients. 2020;12:3004.	Comparator not eligible (multi-component lifestyle intervention, not a diet-only comparison)	Ineligible comparator
Rolland 2009	Rolland C, Hession M, Murray S, Wise A, Broom I. Randomized clinical trial of standard dietary treatment versus a low-carbohydrate/high-protein diet or the LighterLife Programme in the management of obesity. Journal of Diabetes. 2009;1:207–217.	Comparator not eligible (multi-component commercial weight-loss program, not a diet-only comparison)	Ineligible comparator
Samaha 2003	Samaha FF, Iqbal N, Seshadri P, Chicano KL, Daily DA, McGrory J, Williams T, Williams M, Gracely EJ, Stern L. A low-carbohydrate as compared with a low-fat diet in severe obesity. N Engl J Med. 2003;348:2074–2081.	Intervention not isocaloric as specified in the protocol	Energy-matching criterion not met
Santella 2024	Santella B, Mingo M, Papp A, Rice M, Chiappetta S, Calabrese P, Calenda F, Pilone V, Schiavo L. Safety and effectiveness of a 4-week diet on low-carb ready-to-eat ketogenic products as preoperative care treatment in patients scheduled for metabolic and bariatric surgery. Nutrients. 2024;16:3875.	No eligible comparator group	Ineligible comparator
Saslow 2014	Saslow LR, Kim S, Daubenmier JJ, Moskowitz JT, Phinney SD, Goldman V, Murphy EJ, Cox RM, Moran P, Hecht FM. A randomized pilot trial of a moderate carbohydrate diet compared to a very low carbohydrate diet in overweight or obese individuals with type 2 diabetes mellitus or prediabetes. PLoS ONE. 2014;9:e91027.	Intervention not isocaloric as specified in the protocol	Energy-matching criterion not met
Saslow 2017	Saslow LR, Daubenmier JJ, Moskowitz JT, Kim S, Murphy EJ, Phinney SD, Ploutz-Snyder R, Goldman V, Cox RM, Mason AE, Moran P, Hecht FM. Twelve-month outcomes of a randomized trial of a moderate-carbohydrate versus very low-carbohydrate diet in overweight adults with type 2 diabetes mellitus or prediabetes. Nutrition & Diabetes. 2017;7:304.	Intervention not isocaloric as specified in the protocol	Energy-matching criterion not met
Saslow 2017	Saslow LR, Mason AE, Kim S, Goldman V, Ploutz-Snyder R, Bayandorian H, Daubenmier	Intervention not isocaloric as	Energy-matching

	J, Hecht FM. An Online Intervention Comparing a Very Low-Carbohydrate Ketogenic Diet and Lifestyle Recommendations Versus a Plate Method Diet in Overweight Individuals With Type 2 Diabetes: Randomized Controlled Trial. J Med Internet Res. 2017;19(2):e36.	specified in the protocol	criterion not met
Saslow 2023	Saslow LR, Jones LM, Sen A, Wolfson JA, Diez HL, O'Brien A, Leung CW, Bayandorian H, Daubenmier J, Missel AL, Richardson C. Comparing very low-carbohydrate vs DASH diets for overweight or obese adults with hypertension and prediabetes or type 2 diabetes: a randomized trial. Ann Fam Med. 2023;21:256–263.	Comparator not eligible (multi-component lifestyle intervention, not a diet-only comparison)	Ineligible comparator
Schaefer 2005	Schaefer EJ, Gleason JA, Dansinger ML. The effects of low-fat, high-carbohydrate diets on plasma lipoproteins, weight loss, and heart disease risk reduction. Current Atherosclerosis Reports. 2005;7:421–427.	Study design not eligible	Ineligible study design or publication type
Segal-Isaacson 2004	Segal-Isaacson CJ et al. A randomized trial comparing low-fat and low-carbohydrate diets matched for energy and protein. Obesity Research. 2004.	Mechanistic feeding design	Ineligible study design or publication type
Seshadri 2004	Seshadri P, Iqbal N, Stern L, Williams M, Chicano KL, Daily DA, McGrory J, Gracely EJ, Rader DJ, Samaha FF. A randomized study comparing the effects of a low-carbohydrate diet and a conventional diet on lipoprotein subfractions and C-reactive protein levels in patients with severe obesity. American Journal of Medicine. 2004;117:398–405.	Intervention not isocaloric as specified in the protocol	Energy-matching criterion not met
Seshadri 2005	Seshadri P, Samaha FF, Stern L, Chicano KL, Daily DA, Iqbal N. Free fatty acids, insulin resistance, and corrected QT intervals in morbid obesity: effect of weight loss during 6 months with differing dietary interventions. Endocrine Practice. 2005;11(4):234–239.	Intervention not isocaloric as specified in the protocol	Energy-matching criterion not met
Shai 2008	Shai I et al. Weight Loss with a Low-Carbohydrate, Mediterranean, or Low-Fat Diet. N Engl J Med. 2008.	Interventions not isocaloric as specified in the protocol (low-carbohydrate arm non–energy-restricted)	Energy-matching criterion not met
Sharman 2004	Sharman MJ, Gomez AL, Kraemer WJ, Volek JS. Very low-carbohydrate and low-fat diets affect fasting lipids and postprandial lipemia differently in overweight men. Journal of Nutrition. 2004;134:880–885.	The compared dietary interventions were not prescribed with the same energy intake or an	Energy-matching criterion not met

		equivalent target energy deficit.	
Sharman 2004	Sharman MJ, Volek JS. Weight loss leads to reductions in inflammatory biomarkers after a very-low-carbohydrate diet and a low-fat diet in overweight men. Clinical Science. 2004;107:365–369.	The compared dietary interventions were not prescribed with the same energy intake or an equivalent target energy deficit.	Energy-matching criterion not met
Silver 2014	Silver HJ, Kang H, Keil CD, Fazio S, Vaughan DE, Niswender KD. Consuming a balanced high fat diet for 16 weeks improves body composition, inflammation and vascular function parameters in obese premenopausal women. Metabolism. 2014;63:142–152.	Intervention not ketogenic or very low-carbohydrate as specified in the protocol	Ineligible intervention
Singh 2021	Singh M, Hung ES, Cullum A, Allen RE, Aggett PJ, Dyson P, Forouhi NG, Greenwood DC, Pryke R, Taylor R, Twenefour D, Waxman R, Young IS. Lower carbohydrate diets for adults with type 2 diabetes. Diabetic Medicine. 2021;38:e14674.	Study design not eligible	Ineligible study design or publication type
Soenen 2012	Soenen S, Bonomi AG, Lemmens SGT, Scholte J, Thijssen MAMA, van Berkum F, Westerterp-Plantenga MS. Relatively high-protein or "low-carbohydrate" energy-restricted diets for body weight loss and maintenance. Physiology & Behavior. 2012;107:374–380.	Intervention not ketogenic or very low-carbohydrate as specified in the protocol	Ineligible intervention
Sommersten 2022	Sommersten M, Ohlsson C, Svensson M, Karlsson FA, Eliasson B, Smith U, Sjöström CD, Rudling M. Diets differing in carbohydrate cellularity and amount similarly reduced visceral fat in people with obesity (CARBFUNC). Clinical Nutrition. 2022;41:2576–2586.	Intervention not ketogenic or very low-carbohydrate as specified in the protocol	Ineligible intervention
Stern 2004	Stern L et al. The Effects of Low-Carbohydrate versus Conventional Weight Loss Diets in Severely Obese Adults: One-Year Follow-up of a Randomized Trial. Ann Intern Med. 2004	Intervention not isocaloric as specified in the protocol	Energy-matching criterion not met
Struik 2020	Struik NA et al. Very Low and Higher Carbohydrate Diets Promote Differential Appetite Responses in Adults with Type 2 Diabetes: A Randomized Trial. J Nutr. 2020.	Primary outcomes focused on appetite perceptions rather than body weight or body composition as specified in the protocol	Ineligible outcome data
Sun 2023	Sun J et al. Optimal Weight Loss Effect of Short-Term Low Carbohydrate Diet with Calorie Restriction on Overweight/Obese Subjects in South China. BMC Medicine. 2023.	Multi-arm design with combined calorie and carbohydrate	Energy-matching criterion not met

		manipulation; no isoenergetic low-carb vs high-carb comparison	
Tay 2015	Tay J et al. Long-Term Effects of a Very Low Carbohydrate Compared With a High Carbohydrate Diet on Renal Function in Individuals With Type 2 Diabetes: A Randomized Trial. Medicine (Baltimore). 2015;94:e2181.	Linked report from a trial in which achieved carbohydrate intake exceeded the predefined ketogenic threshold.	Ineligible intervention
Tay 2016	Tay J et al. A randomised-controlled trial of the effects of very low-carbohydrate and high-carbohydrate diets on cognitive performance in patients with type 2 diabetes. Br J Nutr. 2016;116:1745–1753.	Linked report from a trial in which achieved carbohydrate intake exceeded the predefined ketogenic threshold.	Ineligible intervention
Tay, 2014	Tay J et al. A very low carbohydrate, low saturated fat diet for type 2 diabetes management: a randomized trial. Diabetes Care. 2014.	Intervention does not meet ketogenic definition after analysis of achieved carbohydrate intake	Ineligible intervention
Tay, 2015	Tay J et al. Comparison of low- and high-carbohydrate diets for type 2 diabetes management: a randomized trial. Am J Clin Nutr. 2015.	Intervention does not meet ketogenic definition after analysis of achieved carbohydrate intake	Ineligible intervention
Tay, 2017	Tay J et al. Effects of an energy-restricted low-carbohydrate, high unsaturated fat diet versus a high-carbohydrate low-fat diet in type 2 diabetes: a 2-year randomized clinical trial. Diabetes Care. 2017.	Intervention does not meet ketogenic definition after analysis of achieved carbohydrate intake	Ineligible intervention
Thomsen 2022	Thomsen MN et al. Dietary carbohydrate restriction augments weight loss-induced improvements in glycaemic control and liver fat in individuals with type 2 diabetes: a randomized controlled trial. Diabetologia. 2022;65:506–517.	Intervention did not meet the predefined ketogenic carbohydrate threshold.	Ineligible intervention
Thomson 2010	Thomson CA et al. Changes in body weight and metabolic indexes in overweight breast cancer survivors enrolled in a randomized trial of low-fat vs reduced-carbohydrate diets. J Am Diet Assoc. 2010.	Intervention not ketogenic or very low-carbohydrate as specified in the protocol	Ineligible intervention
Tirosh 2013	Tirosh A et al. Changes in renal function following three distinct weight loss dietary strategies: A randomized controlled trial (DIRECT). Diabetes Care. 2013.	Intervention not isocaloric as specified in the protocol	Energy-matching criterion not met

Tonstad 2013	Tonstad S et al. A high-fibre bean-rich diet versus a low-carbohydrate diet for obesity. J Hum Nutr Diet. 2013.	Intervention not ketogenic or very low-carbohydrate as specified in the protocol	Ineligible intervention
Torgerson 1999	Torgerson JS et al. Effects on body weight of strict or liberal adherence to an initial period of VLCD treatment. A randomized one-year clinical trial of obese subjects. Int J Obes. 1999;23:190–197.	Intervention not isocaloric as specified in the protocol	Energy-matching criterion not met
Tricò 2021	Tricò D et al. Effects of Low-Carbohydrate versus Mediterranean Diets on Weight Loss, Glucose Metabolism, Insulin Kinetics and β-Cell Function in Morbidly Obese Individuals. Nutrients. 2021.	Intervention not ketogenic or very low-carbohydrate as specified in the protocol	Ineligible intervention
Tsai 2005	Tsai AG et al. Cost-effectiveness of a low-carbohydrate diet and a standard diet in severe obesity. Obesity Research. 2005.	Low-carbohydrate and standard diet interventions were not prescribed with equivalent energy intake or energy deficit.	Energy-matching criterion not met
Umphonsathien 2022	Umphonsathien M et al. Effects of intermittent very-low calorie diet on glycemic control and cardiovascular risk factors in obese patients with type 2 diabetes mellitus. J Diabetes Investig. 2022.	Intervention not isocaloric as specified in the protocol	Energy-matching criterion not met
Valsdottir 2021	Valsdottir TD et al. Low-Carbohydrate High-Fat Diet and Exercise: Effect of a 10-Week Intervention on Body Composition and CVD Risk Factors in Overweight and Obese Women—A Randomized Controlled Trial. Nutrients. 2021.	Intervention not isocaloric as specified in the protocol	Energy-matching criterion not met
Vetter 2010	Vetter ML et al. Effect of a low-carbohydrate diet versus a low-fat, calorie-restricted diet on adipokine levels in obese, diabetic participants. Diabetes Metab Syndr Obes. 2010.	Intervention not isocaloric as specified in the protocol	Energy-matching criterion not met
Veum 2017	Veum VL et al. Visceral adiposity and metabolic syndrome after very high-fat and low-fat isocaloric diets: a randomized controlled trial. Am J Clin Nutr. 2017.	Achieved carbohydrate intake exceeded the predefined ketogenic threshold.	Ineligible intervention
Volek 2004	Volek JS et al. Comparison of a very low-carbohydrate and low-fat diet on fasting lipids, LDL subclasses, insulin resistance, and postprandial lipemic responses in overweight women. J Am Coll Nutr. 2004.	Intervention not isocaloric as specified in the protocol	Energy-matching criterion not met
Wadden 1990	Wadden TA et al. Effects of a very low calorie diet on weight, thyroid hormones and mood. Int J Obes. 1990.	Intervention not isocaloric as	Energy-matching

		specified in the protocol	criterion not met
Wang 2018	Wang L-L et al. The Effect of Low-Carbohydrate Diet on Glycemic Control in Patients with Type 2 Diabetes Mellitus. Nutrients. 2018.	Intervention not ketogenic or very low-carbohydrate as specified in the protocol	Ineligible intervention
Westman 2006	Westman EC et al. Effect of a low-carbohydrate, ketogenic diet program compared to a low-fat diet on fasting lipoprotein subclasses. Int J Cardiol. 2006.	Intervention not isocaloric as specified in the protocol	Energy-matching criterion not met
Westman 2008	Westman EC et al. The effect of a low-carbohydrate, ketogenic diet versus a low-glycemic index diet on glycemic control in type 2 diabetes mellitus. Nutr Metab. 2008;5:36	Intervention not isocaloric as specified in the protocol	Energy-matching criterion not met
Williams 2007	Williams EA et al. Carbohydrate versus Energy Restriction: Effects on Weight Loss, Body Composition and Metabolism. Ann Nutr Metab. 2007.	Non-human study (animal model)	Ineligible population
Wing 1991	Wing RR et al. Effects of a very-low-calorie diet on long-term glycemic control in obese type 2 diabetic subjects. Arch Intern Med. 1991.	Intervention not isocaloric as specified in the protocol (very-low-calorie diet <800 kcal/day)	Energy-matching criterion not met
Wing 1994	Wing RR et al. Year-long weight loss treatment for obese patients with type II diabetes: Does including an intermittent very-low-calorie diet improve outcome? Am J Med. 1994.	Intervention not isocaloric as specified in the protocol	Energy-matching criterion not met
Wycherley 2014	Wycherley TP et al. Long-Term Effects of a Very Low-Carbohydrate Weight Loss Diet on Exercise Capacity and Tolerance in Overweight and Obese Adults. J Am Coll Nutr. 2014.	Secondary report of an already assessed randomized trial; no additional independent participant sample was available.	Duplicate or secondary report
Wycherley 2016	Wycherley TP et al. Long-term effects of weight loss with a very-low carbohydrate, low saturated fat diet on flow mediated dilatation in patients with type 2 diabetes: A randomised controlled trial. Atherosclerosis. 2016.	Secondary publication focusing on endothelial function from an already assessed randomized cohort	Duplicate or secondary report
Wycherley, 2010	Wycherley TP et al. Effects of energy-restricted high-protein, low-fat compared with standard-protein, low-fat diets: a secondary analysis of a randomized trial. Am J Clin Nutr. 2010.	Linked publication from the same cohort reporting overlapping body weight data already represented by Brinkworth et al. 2009.	Duplicate or secondary report

Yancy 2004	Yancy WS Jr et al. A Low-Carbohydrate, Ketogenic Diet versus a Low-Fat Diet To Treat Obesity and Hyperlipidemia. Ann Intern Med. 2004.	Non-isocaloric interventions (energy deficit prescribed only in control arm)	Energy-matching criterion not met
Yancy 2004	Yancy WS et al. A low-carbohydrate diet reduces obesity and may improve dyslipidaemia compared with a low-fat diet. Evid Based Healthc Public Health. 2004.	Secondary publication summarizing an already assessed randomized controlled trial	Duplicate or secondary report
Youssef 2015	Youssef M. The Effect of Dietary Intervention by Low Carbohydrate Diet, and Low Fat Diet, on Weight Loss, Leptin and Adiponectin. Life Sci J. 2015.	Interventions not isocaloric between comparison arms	Energy-matching criterion not met
Zainordin 2021	Zainordin NA et al. Safety and efficacy of very low carbohydrate diet in patients with diabetic kidney disease. PLoS ONE. 2021.	The compared dietary interventions were not prescribed with the same energy intake or an equivalent target energy deficit.	Energy-matching criterion not met
Zinn 2017	Zinn C et al. A 12-week low-carbohydrate, high-fat diet improves metabolic health outcomes over a control diet in a randomised controlled trial with overweight defence force personnel. Appl Physiol Nutr Metab. 2017.	Interventions not isocaloric as specified in the protocol	Energy-matching criterion not met
Zoccali 2023	Zoccali C et al. The effect of a ketogenic diet on weight loss in CKD: a randomized controlled trial in obese stage G1–3a CKD patients. Clin Kidney J. 2023.	Study protocol only – no outcome data available for weight change or body composition	Ineligible study design or publication type

Supplementary Table S5. Study-Level Data Used in the Primary Meta-Analysis

Study	Follow-Up, (weeks)	Ketogenic Diet (n)	Mean Change (kg)	SD of Change (kg)	Higher-Carbohydrate Diet (n)	Mean Change (kg)	SD of Change (kg)	Derivation of SD of Change	Assumed *r*
Alnoubi, 2024	12	14	−10.36	8.13	14	−7.71	6.42	Imputed from baseline and follow-up SDs	0.50
Golay, 1996	6	22	−8.90	2.814	21	−7.50	2.291	Derived from the reported SEM of change	—

Li, 2022	12	24	−8.06	15.04	29	−0.61	14.08	Imputed from baseline and follow-up SDs	0.50
Noakes, 2006	12	24	−8.00	2.94	22	−6.70	3.28	Derived from the reported SEM of change	—
Phillips, 2008	6	10	−5.20	1.897	10	−4.00	1.581	Derived from the reported SE of change	—
Brinkworth, 2009	52	33	−14.50	9.77	36	−11.50	7.20	Derived from the reported SEM of change	—

Values represent complete-case data at the longest eligible follow-up used in the primary meta-analysis. Negative mean changes indicate body-weight reduction.
When the SD of change was not reported, it was derived from a reported SE or SEM or imputed from baseline and follow-up SDs using an assumed within-participant correlation coefficient of r = 0.50.
Sensitivity analyses were conducted using r = 0.25 and r = 0.75 for studies in which SDs of change were imputed from baseline and follow-up SDs.
—, not applicable because the SD of change was derived directly from a reported SE or SEM.
Abbreviations: SD, standard deviation; SE, standard error; SEM, standard error of the mean; r, assumed within-participant correlation between baseline and follow-up measurements.

Supplementary Table S6. Detailed characteristics of dietary interventions and assessment of intervention fidelity.

Study	Feeding setting and food provision	Energy prescription and matching basis	Carbohydrate and protein prescription or achieved intake	Adherence assessment	Ketosis assessment	Energy-matching status	Protein-matching status	Relevant notes
Golay, 1996	Inpatient controlled feeding; all meals prepared according to standardized rotating menus and	Fixed intake of 1000 kcal/day in both groups	LC: 15% energy from CHO, 37 ± 5 g/day; 32% energy from protein. HC: 45% energy	Dietitian present during meals; participants instructed to consume all provided food;	Not assessed	Matched by fixed prescribed intake and controlled feeding	Within protocol-defined tolerance, difference of 3 percentage points of energy	Strongest control of dietary intake among included studies

	consumed under supervision		from CHO, 115 ± 14 g/day; 29% energy from protein	weekly 1-day food records				
Noakes, 2006	Free-living; approximately 36% of total energy supplied as preweighed key foods every 2 weeks	Diets designed to be isoenergetic; approximately 30% energy restriction during weeks 1–8, followed by upward energy adjustment during weeks 9–12 to restore energy balance	VLCARB: target 4% energy from CHO, 35% protein, 61% fat; <20 g CHO/day. VLF: 70% energy from CHO, 20% protein, 10% fat	Daily food checklists; 3-day weighed food records every 2 weeks; fortnightly dietitian consultations; body-weight monitoring; dietary adjustments as required	Plasma ketones	Matched by study design and prescribed energy targets	Not matched, difference of 15 percentage points of energy	Only the VLCARB and VLF groups were eligible for the present review; the third dietary group was not included
Phillips, 2008	Outpatient controlled feeding; all meals prepared and provided by the research center	Identical individualized deficit of 750 kcal/day during weeks 1–4, followed by increased energy intake during the 2-week weight-stabilization phase	LC: Atkins-style diet, 20 g CHO/day; higher protein intake than comparator. LF: AHA-style diet, 30% energy from fat	Food collected every other day; participants reported deviations from the provided diets; dietary intake and physical activity monitored	Daily urinary ketone testing	Matched by identical individualized energy deficit and complete meal provision	Not matched	Reported energy intake was similar between groups within both intervention phases
Tay, 2008; Brinkworth, 2009	Free-living; structured food prescriptions, partial provision of key	Planned isoenergetic intake of approximately 6 MJ/day for women and	VLCHF: 4% energy from CHO, 35% protein, 61% fat; <20 g CHO/day	Quantitative daily food records; food weighing; 3-day	Plasma ketones measured repeatedly during	Matched by planned isoenergetic prescriptions; reported energy intake was	Not matched, difference of 11 percentage points of energy	The two reports represent the same randomized cohort at 24 and 52

	foods during the first 8 weeks, followed by food vouchers	7 MJ/day for men, corresponding to an approximately 30% energy deficit	during weeks 1–8, with allowance up to <40 g/day thereafter. HCLF: 46% energy from CHO, 24% protein, 30% fat	records analyzed within consecutive 2-week periods; frequent dietitian consultations; body-weight monitoring	follow-up	similar between groups		weeks; the 52-week report was used in the meta-analysis
Ruth, 2013	Free-living; food not provided	Identical individualized deficit of 500 kcal/day in both groups, estimated using the Mifflin–St Jeor equation	HFLC prescribed: ≤40 g CHO/day, approximately 35% energy from protein. Achieved: 39.4 ± 59.5 g CHO/day, 9.6 ± 7.7% energy from CHO, 33.5 ± 7.9% protein. LFHC prescribed: approximately 60% energy from CHO and 15% protein. Achieved: 201.8 ± 83.0 g CHO/day, 55.7 ± 12.6% energy from CHO, 22.0 ± 5.4% protein	Registered dietitian consultations every 2 weeks; 3-day food records completed every 2 weeks	Not assessed	Matched by identical prescribed energy deficit; achieved energy intake was similar between groups	Not matched	Achieved energy intake: HFLC 1532 ± 468 kcal/day; LFHC 1439 ± 485 kcal/day. High post-randomization attrition

Li, 2022	Outpatient dietary intervention; full food provision not reported	1500 ± 50 kcal/day prescribed in both groups	KD: 30–50 g CHO/day, 60 g protein/day, 130 g fat/day. Control: 250–280 g CHO/day, 60 g protein/day, 20 g fat/day. Achieved macronutrient intake NR	Three face-to-face preparatory sessions and nutrition education before the intervention; detailed quantitative monitoring during follow-up NR	Not clearly reported	Matched by identical prescribed energy intake; achieved energy intake NR	Matched by prescription, 60 g/day in both groups	Adherence reporting was limited; long-term acceptability of the KD was described as lower than that of the routine diabetes diet
Alnoubi and Alqurashi, 2024	Free-living; weekly meal plans and dietary counselling; food not fully provided	Equal individualized total energy prescribed in both groups; requirements estimated from basal metabolic rate and activity; numerical energy targets and achieved intake NR	HFKD: 10% energy from CHO, 20% protein, 70% fat. LFD: 55% energy from CHO, 25% protein, 20% fat. Achieved quantitative intake NR	Daily food photographs and food lists; weekly meal plans and counselling; authors reported >95% adherence to macronutrient targets	Urinary ketones assessed with reagent strips	Matched by prescribed individualized energy intake; achieved energy intake NR	Within protocol-defined tolerance, difference of 5 percentage points of energy	Participants were advised to maintain 8000–10,000 steps/day, monitored with smartwatches

Study identifiers correspond to the references listed in the main manuscript.

Energy matching refers to equivalence of the prescribed total energy intake or target energy deficit between intervention groups. It does not necessarily indicate that objectively measured or self-reported energy intake remained identical throughout follow-up.

Protein matching was assessed according to the criterion specified in the registered protocol: a between-group difference of no more than 0.3 g/kg/day or no more than 5 percentage points of total energy.

Where prescribed and achieved dietary intake were both available, they are distinguished within the same cell. No achieved intake values were inferred when they were not explicitly reported.

Abbreviations: AHA, American Heart Association; CHO, carbohydrate; HC, higher-carbohydrate diet; HCLF, high-carbohydrate low-fat diet; HFLC, high-fat low-carbohydrate diet; HFKD, high-fat ketogenic diet; KD, ketogenic diet; LC, low-carbohydrate diet; LF, low-fat diet; LFHC, low-fat high-carbohydrate diet; LFD, low-fat diet; NR, not reported; VLF, very-low-fat diet; VLCARB, very-low-carbohydrate diet; VLCHF, very-low-carbohydrate high-fat diet.

Supplementary Table S7. Risk-of-bias assessments for the body-weight outcome using the RoB 2 tool.

Study	D1	D2	D3	D4	D5	Overall	Rationale
Brinkworth 2009	Low risk	Low risk	Some concerns	Low risk	Some concerns	Some concerns	Random sequence generation and allocation concealment were adequately described in reports from the same trial cohort. No relevant deviations from the assigned interventions were identified. Body-weight data at 52 weeks were unavailable for a substantial proportion of randomized participants. Attrition was broadly comparable between groups, but an association between missingness and the true body-weight outcome could not be excluded. Body weight was measured objectively. Some concerns remained regarding selection of the reported result because no sufficiently detailed prespecified analysis plan for body-weight change was identified.
Ruth 2013	Some concerns	Low risk	Some concerns	Low risk	Some concerns	Some concerns	Block randomization was reported, but allocation concealment was insufficiently described. No relevant deviations from the assigned interventions were identified. Body-weight data were unavailable for 22 of 55 randomized participants. Attrition was broadly balanced between groups, and the authors stated that an intention-to-treat analysis produced conclusions consistent with the completer analysis; however, complete numerical results were not reported. Body weight was measured objectively. Some concerns remained regarding selection of the reported result because no prospective protocol or detailed statistical analysis plan was identified.
Golay 1996	Some concerns	Low risk	Low risk	Low risk	Some concerns	Some concerns	Random allocation was reported, but sequence generation and allocation concealment were insufficiently described. The six-week intervention was conducted in a controlled-

							feeding setting with complete provision and supervision of the isoenergetic diets, and no relevant deviations from the assigned interventions were reported. Body-weight data were complete and were measured objectively. Some concerns remained regarding selection of the reported result because no prospective protocol or prespecified statistical analysis plan was identified.
Noakes 2006	Some concerns	Low risk	Some concerns	Low risk	Some concerns	Some concerns	Participants were randomly allocated after matching for age, sex, and BMI, but sequence generation and allocation concealment were insufficiently described. Dietary interventions were delivered and monitored similarly across groups, and no relevant deviations arising from the trial context were identified. The body-weight result used in the review was based on participants with available follow-up data. Attrition was not markedly imbalanced between groups, but dependence of missingness on adherence or the true body-weight outcome could not be excluded. Body weight was measured objectively. Some concerns remained regarding selection of the reported result because no prospective protocol or sufficiently detailed statistical analysis plan was identified.
Li 2022	Some concerns	Some concerns	High risk	Low risk	Some concerns	High risk	Random allocation was reported, but sequence generation and allocation concealment were insufficiently described. Several participants discontinued the ketogenic diet because they were unable to adhere to the assigned intervention, and the analysis did not fully preserve the effect of assignment. Body-weight data were unavailable for 6 of 30 participants in the ketogenic group and 1 of 30 in the control group. Because attrition was markedly imbalanced and related to tolerability of the assigned diet, missingness

							could plausibly depend on the true body-weight outcome. Body weight was measured objectively. Some concerns remained regarding selection of the reported result because no prospective protocol or detailed statistical analysis plan was identified.
Phillips 2008	Some concerns	Some concerns	Some concerns	Low risk	Some concerns	Some concerns	Random allocation was reported, but sequence generation and allocation concealment were insufficiently described. Information was insufficient to exclude differential deviations from the assigned interventions, and the analysis did not include all randomized participants. Twenty of 28 randomized participants were included in the reported body-weight analysis. Reasons for post-randomization exclusion were reported, but the possibility that missing outcome data were related to the true body-weight outcome could not be excluded. Body weight was measured objectively. Some concerns remained regarding selection of the reported result because no prospective protocol or detailed statistical analysis plan was identified and body weight was not the principal outcome of the trial.
Alnoubi 2024	Low risk	Low risk	Low risk	Low risk	Some concerns	Some concerns	The randomization schedule was generated using a centrally controlled computerized system, with allocation concealed until enrollment. Dietary support, monitoring, and physical-activity recommendations were applied similarly across groups, and no relevant deviations from the assigned interventions were identified. Body-weight data were available for all randomized participants and were measured objectively under standardized conditions. Some concerns remained regarding selection of the reported result because trial registration occurred after recruitment had begun, no detailed prespecified

statistical analysis plan was available, and multiple eligible approaches to analyzing body-weight change were possible.

Assessments refer to the effect of assignment to intervention on change in body weight.

D1, bias arising from the randomization process; D2, bias due to deviations from intended interventions; D3, bias due to missing outcome data; D4, bias in measurement of the outcome; D5, bias in selection of the reported result; RoB 2, revised Cochrane risk-of-bias tool for randomized trials.

Supplementary Figures

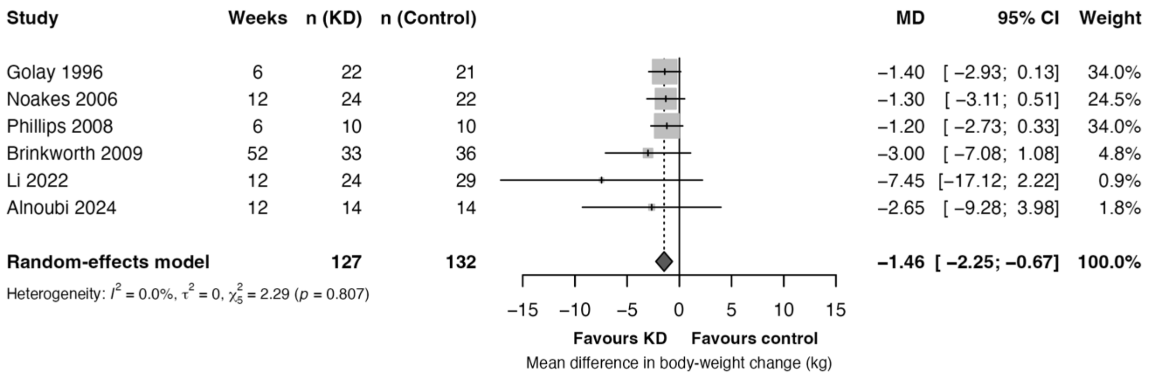

Supplementary Figure S1. Forest plot of the sensitivity analysis assuming a within-participant correlation coefficient of r = 0.25.

Standard deviations of change that were unavailable in the original reports were imputed from baseline and follow-up standard deviations assuming r = 0.25. The pooled mean difference was estimated using a random-effects model with restricted maximum-likelihood estimation and the Hartung–Knapp adjustment. Negative mean differences indicate greater body weight loss in the ketogenic diet group. CI, confidence interval; KD, ketogenic diet; MD, mean difference; SD, standard deviation.

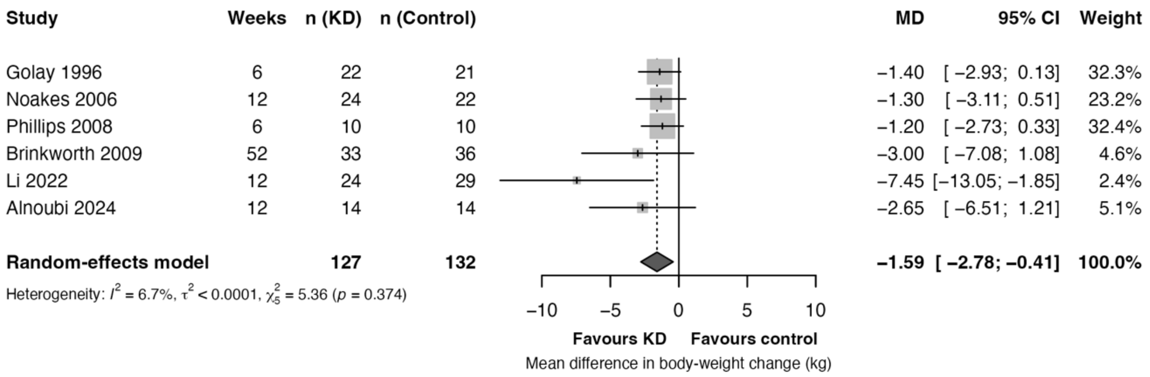

Supplementary Figure S2. Forest plot of the sensitivity analysis assuming a within-participant correlation coefficient of r = 0.75.

Standard deviations of change that were unavailable in the original reports were imputed from baseline and follow-up standard deviations assuming r = 0.75. The pooled mean difference was estimated using a random-effects model with restricted maximum-likelihood estimation

and the Hartung–Knapp adjustment. Negative mean differences indicate greater body weight loss in the ketogenic diet group. CI, confidence interval; KD, ketogenic diet; MD, mean difference; SD, standard deviation.

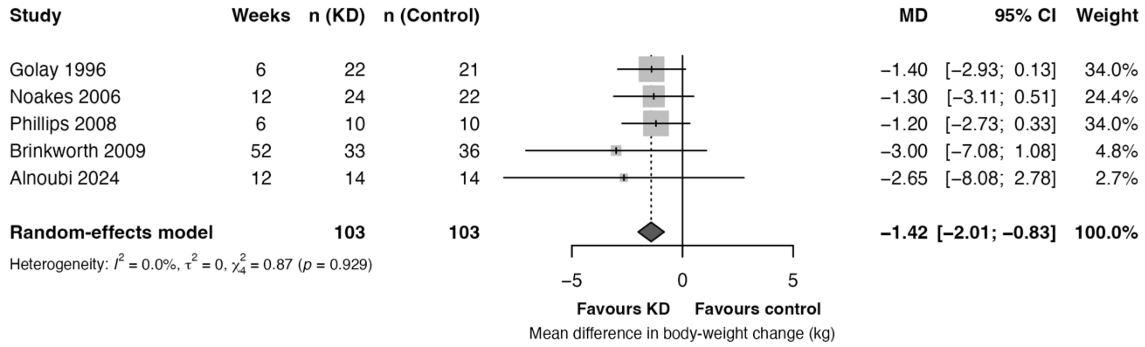

Supplementary Figure S3. Forest plot of the sensitivity analysis excluding the study rated as being at overall high risk of bias.

Li, 2022 was excluded because it was rated as being at overall high risk of bias according to the RoB 2 assessment. The pooled mean difference was estimated using a random-effects model with restricted maximum-likelihood estimation and the Hartung–Knapp adjustment. Negative mean differences indicate greater body weight loss in the ketogenic diet group. CI, confidence interval; KD, ketogenic diet; MD, mean difference; RoB 2, revised Cochrane risk-of-bias tool for randomized trials.

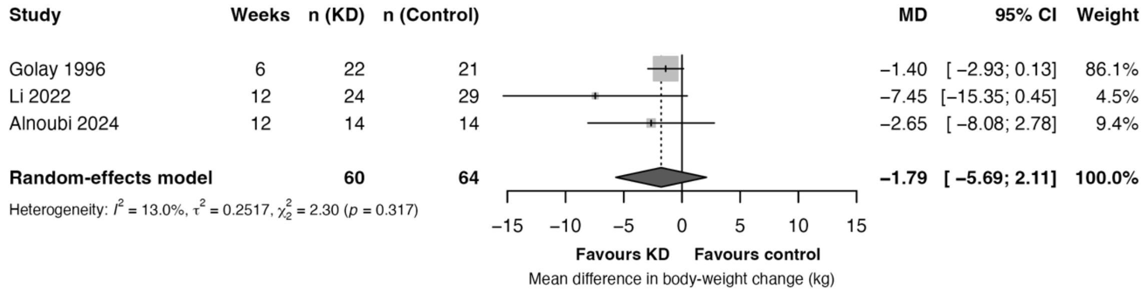

Supplementary Figure S4. Forest plot of the exploratory sensitivity analysis restricted to studies meeting the prespecified protein-matching criterion.

The analysis included studies in which the between-group difference in prescribed protein intake did not exceed 0.3 g/kg/day or 5 percentage points of total energy, as specified in the registered protocol. The pooled mean difference was estimated using a random-effects model with restricted maximum-likelihood estimation and the Hartung–Knapp adjustment. Negative mean differences indicate greater body weight loss in the ketogenic diet group. CI, confidence interval; KD, ketogenic diet; MD, mean difference.

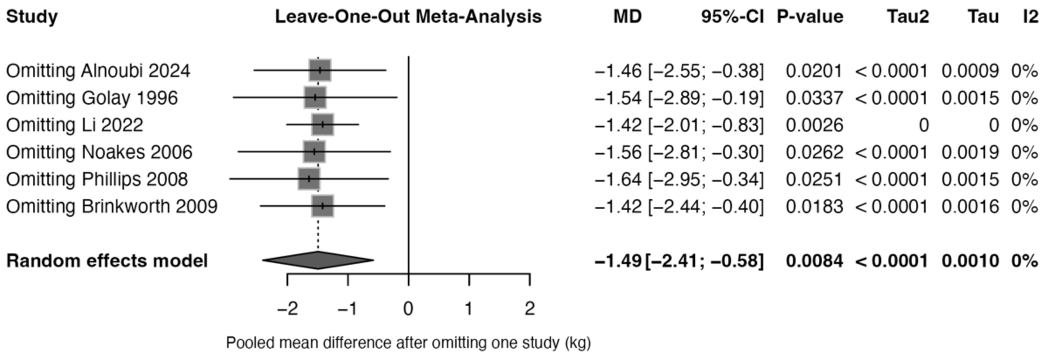

Supplementary Figure S5. Leave-one-out sensitivity analysis of the pooled effect on body weight loss.

Each row shows the pooled mean difference obtained after excluding the indicated study. The estimate based on all six studies represents the primary meta-analysis. Estimates were calculated using random-effects models, with between-study variance estimated using restricted maximum likelihood and confidence intervals calculated using the Hartung–Knapp adjustment. Negative mean differences indicate a greater reduction in body weight in the ketogenic diet group. CI, confidence interval; I^2, proportion of total variability attributable to between-study heterogeneity; MD, mean difference; p, p-value for the pooled effect; τ, estimated between-study standard deviation; τ^2, estimated between-study variance.
